# Claudin-10 Expression and the Gene Expression Pattern of Thick Ascending Limb Cells

**DOI:** 10.3390/ijms25074008

**Published:** 2024-04-03

**Authors:** Gaelle Brideau, Lydie Cheval, Camille Griveau, Wung-Man Evelyne Ling, Loïc Lievre, Gilles Crambert, Dominik Müller, Jovana Broćić, Emeline Cherchame, Pascal Houillier, Caroline Prot-Bertoye

**Affiliations:** 1 Centre de Recherche des Cordeliers, Institut National de la Santé et de la Recherche Médicale, Sorbonne Université, Université Paris Cité, F-75006 Paris, France; gaelle.brideau@crc.jussieu.fr (G.B.); lydie.cheval@crc.jussieu.fr (L.C.); camille_griveau@orange.fr (C.G.); ling_evelyne@hotmail.fr (W.-M.E.L.); llievre@chu-reims.fr (L.L.); gilles.crambert@crc.jussieu.fr (G.C.); 2 Centre National de la Recherche Scientifique, Equipe Mixte de Recherche 8228-Laboratoire de Physiologie Rénale et Tubulopathies, F-75006 Paris, France; 3 Department of Pediatrics, Division of Gastroenterology, Nephrology and Metabolic Diseases, Charité-Universitätsmedizin Berlin, DE-13353 Berlin, Germany; dominik.mueller@charite.de; 4 Paris Brain Institute (ICM), Hôpital Pitié-Salpêtrière, Inserm U 1127, CNRS UMR 7225, Sorbonne Université, Data Analysis Core Platform, F-75013 Paris, France; jovana.brocic@icm-institute.org (J.B.); emeline.cherchame@icm-institute.org (E.C.); 5 Assistance Publique-Hôpitaux de Paris, Hôpital Européen Georges Pompidou, Service de Physiologie, F-75015 Paris, France; 6 Centre de Référence des Maladies Rénales Héréditaires de l’Enfant et de l’Adulte (MARHEA), The European Rare Kidney Disease Reference Network (ERKNet), F-75015 Paris, France; 7 Centre de Référence des Maladies Rares du Calcium et du Phosphate, The European Reference Network on Rare Endocrine Conditions (Endo-ERN), F-75015 Paris, France; 8 Faculté de Médecine, Université Paris Cité, F-75006 Paris, France

**Keywords:** tight junction, epithelium, kidney, claudin-10, HELIX syndrome, thick ascending limb of the loop of Henle, transcriptional profiling

## Abstract

Many genomic, anatomical and functional differences exist between the medullary (MTAL) and the cortical thick ascending limb of the loop of Henle (CTAL), including a higher expression of claudin-10 (CLDN10) in the MTAL than in the CTAL. Therefore, we assessed to what extent the *Cldn10* gene expression is a determinant of differential gene expression between MTAL and CTAL. RNAs extracted from CTAL and MTAL microdissected from wild type (WT) and Cldn10 knock out mice (cKO) were analyzed by RNAseq. Differential and enrichment analyses (GSEA) were performed with interactive R Shiny software. Between WT and cKO MTAL, 637 genes were differentially expressed, whereas only 76 were differentially expressed between WT and cKO CTAL. Gene expression patterns and GSEA analyses in all replicates showed that WT MTAL did not cluster with the other replicates; no hierarchical clustering could be found between WT CTAL, cKO CTAL and cKO MTAL. Compared to WT replicates, cKO replicates were enriched in *Cldn16*, *Cldn19*, *Pth1r*, (parathyroid hormone receptor type 1), *Casr* (calcium sensing receptor) and *Vdr* (Vitamin D Receptor) mRNA in both the cortex and medulla. *Cldn10* is associated with gene expression patterns, including genes specifically involved in divalent cations reabsorption in the TAL.

## 1. Introduction

The thick ascending limb of the loop of Henle (TAL) plays an important role in water, sodium chloride (NaCl), acid and divalent cation homeostasis in ground-dwelling mammals [1,2]. It reabsorbs more than half of filtered magnesium (Mg^2+^); about one quarter of filtered NaCl, bicarbonate and calcium (Ca^2+^); and the major part of ammonium that is secreted in the proximal tubule [1,2]. Accordingly, monogenic diseases that affect ion transport in the TAL commonly have severe consequences. Bartter syndrome (OMIM #601678, #241200, #607364, #602522, #300971) is a rare inherited salt-losing tubulopathy caused by impaired transcellular NaCl reabsorption along the TAL, linked to different genetic variants (*SLC12A1*, *KCNJ1*, *CLCNKA*, *CLCNKB*, *BSND*, *MAGED2)* that encode, respectively, the Na^+^-K^+^-2Cl^−^ cotransporter type 2 NKCC2, the potassium channel ROMK, the chloride channels ClC-Ka and ClC-Kb, the chloride channel subunit Barttin and MAGE-D2 [3]. Another cause of salt-losing tubulopathy stemming from the TAL is HELIX syndrome (hypohidrosis, electrolyte disturbances, hypolacrimia, ichthyosis, xerostomia, OMIM #617671), due to biallelic loss-of-function variants of claudin-10b (CLDN10B); under this condition, the transcellular transport of NaCl is not decreased, however, the paracellular transport is altered because the relative paracellular sodium to chloride permeability ratio is low [4,5,6,7,8,9,10]. FHHNC (Familial Hypomagnesemia with Hypercalciuria and Nephrocalcinosis (OMIM #248250, #248190) is another condition characterized by a defect in paracellular ion transport in the TAL that causes a massive loss of Ca^2+^ and Mg^2+^ in urine but no loss of NaCl [11,12,13,14]. It is causally linked to biallelic variants of claudin-16 (CLDN16) and claudin-19 (CLDN19) [11,12,13,14].

The TAL shows a higher degree of functional heterogeneity than originally assumed [1]. We have known for many years that ion transport can occur along a transcellular (Na, Cl) or a paracellular (Na, Cl, Mg, Ca) route [1,2]. What is new is the demonstration that the permeability properties of the paracellular pathway vary according to the claudin(s) expressed at the tight junction [15]: the expression of CLDN10B increases the sodium to chloride permeability ratio, whereas the expression of both CLDN16 and CLDN19 is required for a high paracellular permeability to Ca^2+^ and Mg^2+^ [9,10,11,13,16,17]. The TAL shows also a significant axial heterogeneity [1,2]. Regarding the transcellular transport of ions for example, the distinct isoforms of NKCC2 are involved in NaCl reabsorption in the medullary and cortical part of the TAL [1,18,19]. Chloride channel CLCK1, the murine analog of CLCKA, is expressed only in the medullary TAL whereas CLCK2, the murine analog of CLCKB, is expressed along the entire TAL [2,20]. Concerning the paracellular transport of ions, most of the Ca^2+^ and Mg^2+^ reabsorption takes place in the cortical TAL (CTAL), with almost no Ca^2+^ and Mg^2+^ being absorbed in the inner part of the outer medulla (ISOM) [21,22,23]. Accordingly, CLDN16 expression is higher in the cortex than in the medulla, with the converse being true regarding CLDN10 expression [24,25,26]. Recent single cell transcriptomic analyses indicate that at least two CTAL cell types express either *Cldn16* or *Cldn10* [27,28]. It should be pointed out that there are two types of tight junctions in the CTAL, the first expressing CLDN10 and the second expressing CLDN16-CLDN19 [29,30]. Both are expressed in the CTAL, whereas only one type of tight junction expressing CLDN10 is found in the ISOM TAL [29,30,31]. When CLDN10 is lacking, the mosaic pattern of CLDN expression is abolished: CLDN16 is expressed in all tight junctions and CLDN19 in almost all tight junctions in the CTAL [29]. Moreover, CLDN16 expression is extended to the tight junctions in ISOM TAL [10]. Then, it appears that CLDN10 expression is an important determinant of the properties of medullary and cortical paracellular pathway in the TAL. Whether its expression determines more deeply the phenotype of TAL cells, either cortical or medullary, is unknown. In order to fill this gap in knowledge, we compared the transcriptome of cortical and medullary TAL (CTAL and MTAL, respectively) cells from normal mice and mice with a depletion in CLDN10 expression [9].

## 2. Results

Five pools of wild type (WT) CTALs, five pools of WT MTALs, five pools of *Cldn10* conditional knock-out (cKO) CTALs and three pools of cKO MTALs were used for analysis. Indeed, cKO MTAL dissection was made extremely difficult due to nephrocalcinosis present in the outer medulla of cKO kidney sections and only three replicates passed quality control [9]. We confirmed a lower expression but not a total deletion of *Cldn10* in cKO CTAL and MTAL than in WT CTAL and MTAL (Figure S1) as we used a conditional *Cldn10*-deficient mouse model [9]. A high expression of *Slc12a1* (encoding NKCC2) and a low expression of *Slc2a2* (encoding the sodium–glucose cotransporter 2 expressed in the proximal tubule [24]), of *Slc12a3* (encoding the thiazide sensitive Na^+^–Cl^−^ cotransporter NCC expressed in the distal convoluted tubule [24]) and of *Aqp2* (Aquaporin-2, expressed in the connecting tubule and the collecting duct [24]) confirmed specific TAL dissection without significant contamination by the proximal tubule, distal convoluted tubule or collecting duct (Figure S1).

### 2.1. Differences between cKO MTAL and WT MTAL Transcriptomes

Since *Cldn10* expression is the highest in WT MTAL (Figure S1), we first examined the effect of its deletion in MTAL.

A total of 637 genes were differentially expressed between WT MTAL and cKO MTAL replicates, whereas the expression of 11,393 genes did not differ between both groups (using a log2 foldchange threshold of 1 and a corrected *p*-value (FDR) < 0.05). Compared to WT MTAL replicates, 179 genes were less expressed and 458 genes more expressed in *cKO* MTAL replicates (Figure S2, Table S1).

**Figure 1:**
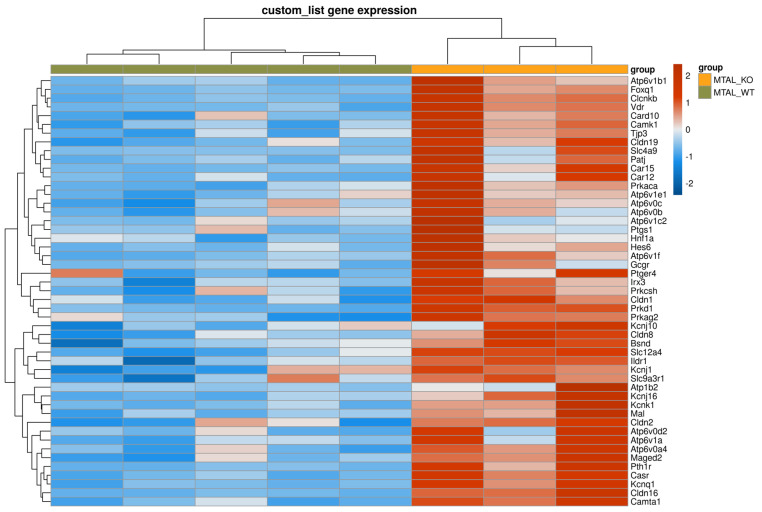
Supervised clustering of cKO MTAL and WT MTAL based on a list of genes with likely preferential expression in the TAL. The heatmap was generated using the pheatmap R package on three pools of cKO MTAL and five pools of WT MTAL. Clustering was constructed on 212 genes using Euclidean methods and complete linkage. The gradient of colors represents the expression level (z-score).

**Figure 2:**
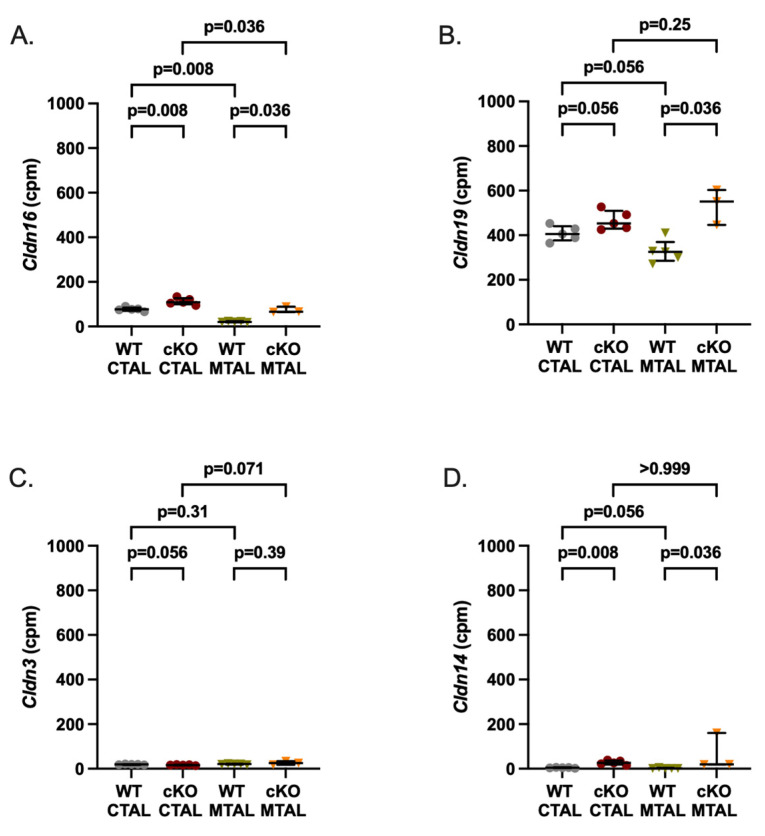
Gene expression analysis of claudins (Cldn) in WT and cKO MTAL and CTAL. (**A**) *Cldn16*. (**B**) *Cldn19*. (**C**) *Cldn3*. (**D**) *Cldn14*. Medians and interquartile ranges are shown (Mann–Whitney test); counts per millions (cpm).

**Figure 3:**
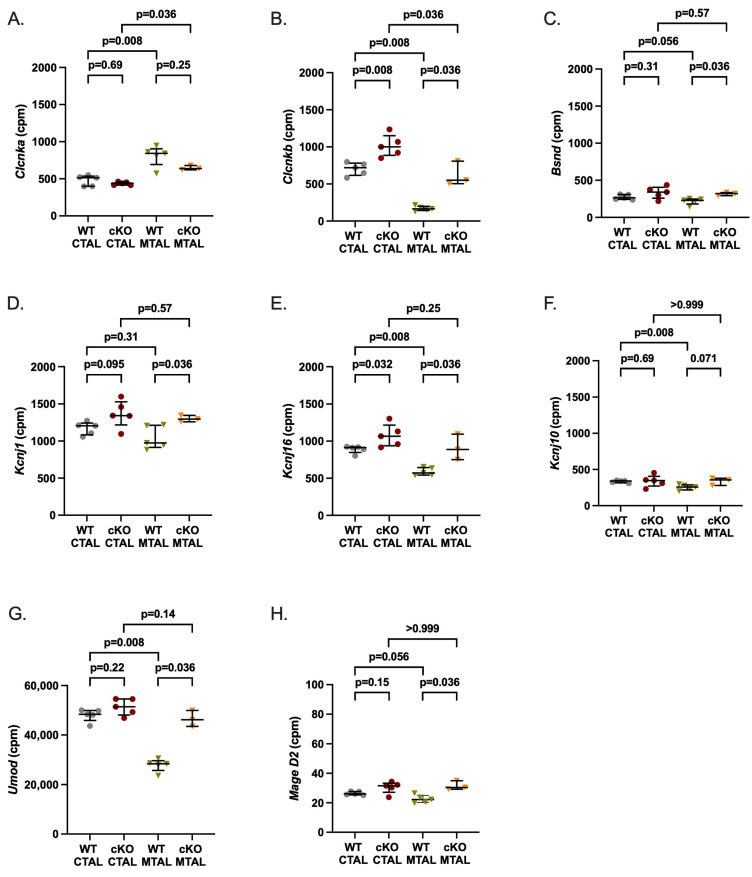
Gene expression analysis of specific ions channels, MageD2 and uromodulin in WT and cKO MTAL and CTAL. (**A**) *Clcnka* encodes the chloride channel ClC-Ka. (**B**) *Clcnkb* encodes the chloride channel ClC-Kb. (**C**) *Bsnd* encodes Barttin, an essential subunit for ClC chloride channel. (**D**) *Kcnj1* encodes the potassium channel ROMK. (**E**) *Kcnj16* encodes the potassium channel KIR5.1. (**F**) *Kcnj10* encodes the potassium channel KIR4.1. (**G**) *Umod* (uromodulin). (**H**) *MageD2*. The scales are different for *Umod* and *MageD2*. Medians and interquartile ranges are shown (Mann–Whitney test); counts per millions (cpm).

**Figure 4:**
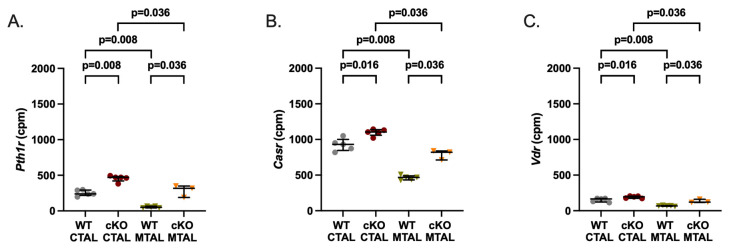
Gene expression analysis of receptors in WT and cKO MTAL and CTAL. (**A**) Parathyroid hormone receptor type 1 (*Pth1r)*. (**B**) Calcium-sensing receptor *(Casr)*. (**C**) Vitamin D receptor (*Vdr)*. Medians and interquartile ranges are shown (Mann–Whitney test); counts per millions (cpm).

We analyzed the data from the custom list of 222 genes with a likely preferential expression in the TAL (Table S2); compared to WT MTAL replicates, cKO MTAL replicates were enriched in 49 genes, including *Cldn16*, *Cldn19*, *Pth1r* (parathyroid hormone receptor type 1), *Casr* (calcium sensing receptor) and *Vdr* (Vitamin D receptor) (Figure 1). Phosphatases, kinases (including *Prkag1* (protein kinase, AMP-activated, gamma 1 non-catalytic subunit), *Prkag2* (protein kinase, AMP-activated, gamma 2 non-catalytic subunit), *Prkaca* (protein kinase, cAMP-dependent, catalytic, alpha)), transcription factors (including *Foxq1* (forkhead box Q1) and *Irx3* (Iroquois-related homeobox 3)) gene expression (Figures S3–S5) allowed a hierarchical clustering of all cKO and WT MTAL replicates. *Cldn16*, *Cldn19*, *Cldn14*, *Clcnkb*, *Bsnd* (barttin), *Kcnj1* (potassium channel ROMK), *Kcnj16*, *Umod* (Uromodulin), *MageD2*, *Pth1r*, *Casr* and *Vdr* were more expressed in cKO MTAL than in WT MTAL (Figure 2, Figure 3 and Figure 4).

### 2.2. Differences between cKO CTALs and WT CTALs Transcriptomes

A total of 76 genes were differentially expressed between WT CTAL and cKO CTAL replicates, whereas the expression of 11,403 genes did not differ between both groups. Compared to WT CTAL replicates, 29 genes were less expressed and 37 genes were more expressed in cKO CTAL replicates (Table S3, Figure S6).

**Figure 5:**
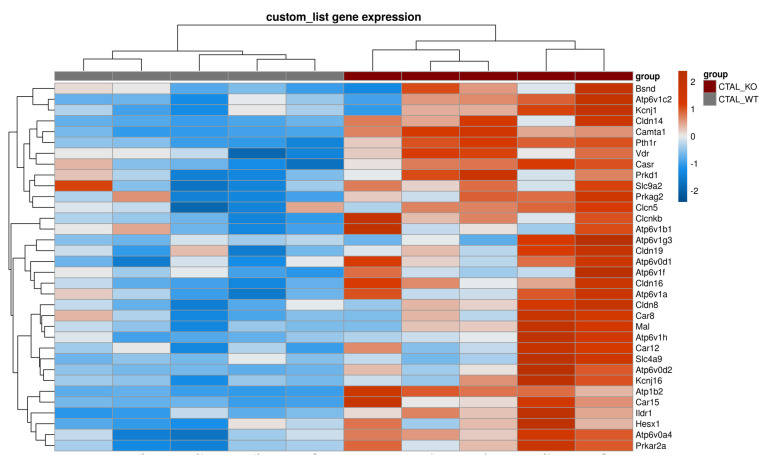
Supervised clustering of cKO CTAL and WT CTAL based on a list of genes with a likely preferential expression in the TAL. The heatmap was generated using pheatmap R package on five pools of cKO CTAL and five pools of WT CTAL. Clustering was constructed on 212 genes using Euclidean methods and complete linkage. The gradient of colors represents the expression level (z-score).

The GSEA analysis of the custom list of 222 genes (Table S2) revealed that cKO CTAL were enriched in 34 genes, as compared to WT CTAL (Figure 5). Phosphatases, kinases and transcription factors expression did not allow a hierarchical clustering of all cKO and WT CTAL replicates.

*Cldn16*, *Cldn14*, *Clcnkb*, *Kcnj16*, *Pth1r*, *Casr* and *Vdr* were more expressed in cKO CTAL than in WT CTAL (Figure 2, Figure 3 and Figure 4).

### 2.3. Differences between WT MTAL and WT CTAL Transcriptomes

A total of 291 genes were differentially expressed between WT MTAL and WT CTAL replicates, whereas the expression of 11,348 genes did not differ between both groups. Compared to WT MTAL replicates, 205 genes were more expressed and 86 genes were less expressed in WT CTAL replicates (Figure S7, Table S4).

**Figure 6:**
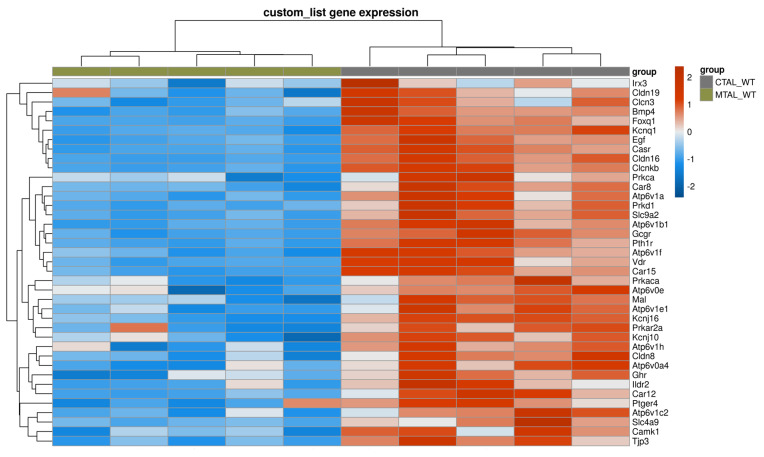
Supervised clustering of WT MTAL and WT CTAL based on a list of genes with a likely preferential expression in the TAL. The heatmap was generated using pheatmap R package on five pools of WT CTAL and five pools of WT MTAL. Clustering was constructed on 217 genes using Euclidean methods and complete linkage. The gradient of colors represents the expression level (z-score).

The GSEA analysis of the custom list of 222 genes (Table S2) showed that WT CTAL was enriched in 39 genes, compared to WT MTAL, including *Cldn16*, *Cldn19*, *Pth1r*, *Casr* and *Vdr* (Figure 6). The expression of phosphatases, kinases (including *Prkag2* (protein kinase, AMP-activated, gamma 2 non-catalytic subunit), *Prkaca* (protein kinase, cAMP-dependent, catalytic, alpha)) and transcription factors (including *Foxq1* (forkhead box Q1) and *Irx3* (Iroquois-related homeobox 3)) (Figures S8–S10) allowed a hierarchical clustering of all WT MTAL and CTAL replicates.

*Cldn10* was more expressed and *Cldn16* was less expressed in WT MTAL than in WT CTAL while the expression of *Cldn19*, *Cldn14* and *Cldn3* did not differ between groups (Figure S1 and Figure 2). Regarding transcellular ion channels, *Clcnka* was more expressed in MTAL than in CTAL, whereas it was the converse for *Clcnkb* and the potassium channels *Kcnj10* and *Kcnj16* (Figure 3). The expression of *Bsnd*, *Kcnj1* and *MageD2* was similar in WT MTAL and CTAL (Figure 3). *Umod* was less expressed in WT MTAL than in WT CTAL (Figure 3). *Pth1r*, *Casr* and *Vdr* were significantly less expressed in WT MTAL than in WT CTAL (Figure 4).

### 2.4. Differences between cKO MTAL and cKO CTAL Transcriptomes

**Figure 7:**
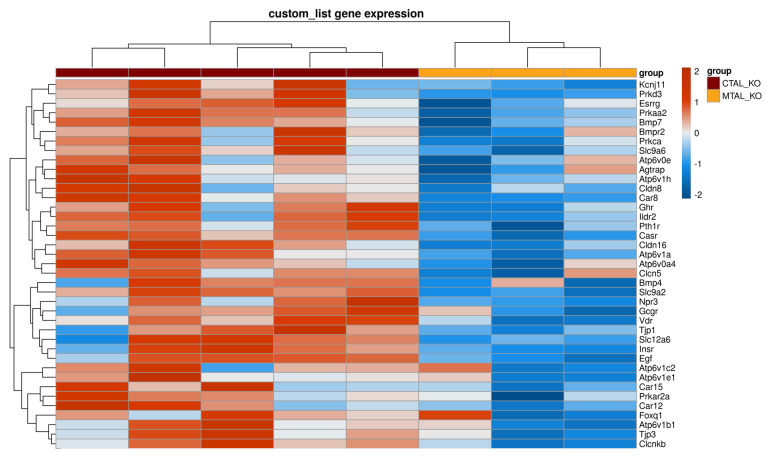
Supervised clustering of cKO MTAL and cKO CTAL based on a list of genes with a likely preferential expression in the TAL. The heatmap was generated using a pheatmap R package on five pools of cKO CTAL and three pools of cKO MTAL. Clustering was constructed on 211 genes using Euclidean methods and complete linkage. The gradient of colors represents the expression level (z-score).

A total of 359 genes were differentially expressed between cKO MTAL and cKO CTAL replicates, whereas the expression of 11,664 genes was similar between groups (Figure S11, Table S5). Unsupervised RNA-seq analysis showed that cKO MTALs and cKO CTALs did not cluster differentially (Figure S11). The GSEA analysis of the custom list of 222 genes showed that compared to cKO MTAL replicates, the cKO CTAL replicates were enriched in 39 genes, including *Cldn16*, *Pth1r*, *Casr* and *Vdr* (Figure 7). Phosphatase and transcription factor (including *Irx3* (Iroquois-related homeobox 3)) expression allowed a hierarchical clustering of all cKO MTAL and cKO CTAL replicates (Figures S12 and S13).

*Cldn16* was less expressed in cKO MTAL than in cKO CTAL (Figure 2). *Clcnka* and *Clcnkb* were more and less expressed in cKO MTAL than in cKO CTAL, respectively (Figure 3). The expressions of *Bsnd*, *Kcnj1*, *Kcnj10*, *Kcnj16*, *Umod* and *MageD2* were similar in both cKO MTAL and cKO CTAL (Figure 3). *Pth1r*, *Casr* and *Vdr* were significantly less expressed in cKO MTAL than in cKO CTAL (Figure 4).

### 2.5. Differential Transcriptomes of cKO MTAL, WT MTAL, cKO CTAL and WT CTAL

**Figure 8:**
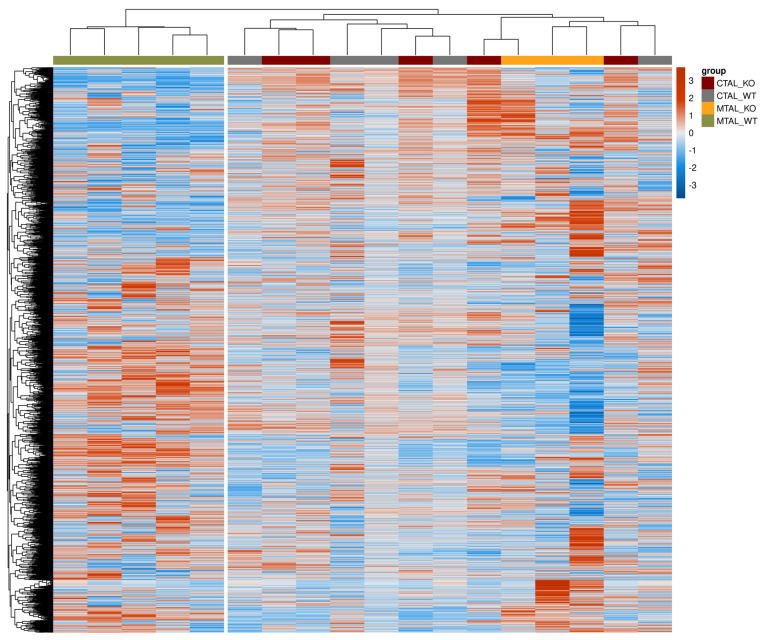
Heatmap showing gene expression pattern for all replicates of cKO MTAL, WT MTAL, cKO CTAL and WT CTAL. The heatmap was generated using pheatmap R package on five pools of WT CTAL, five pools of cKO CTAL, five pools of WT MTAL and three pools of cKO MTAL. Clustering was constructed on 10,548 genes using correlation methods and complete linkage. The gradient of colors represents the expression level (z-score).

**Figure 9:**
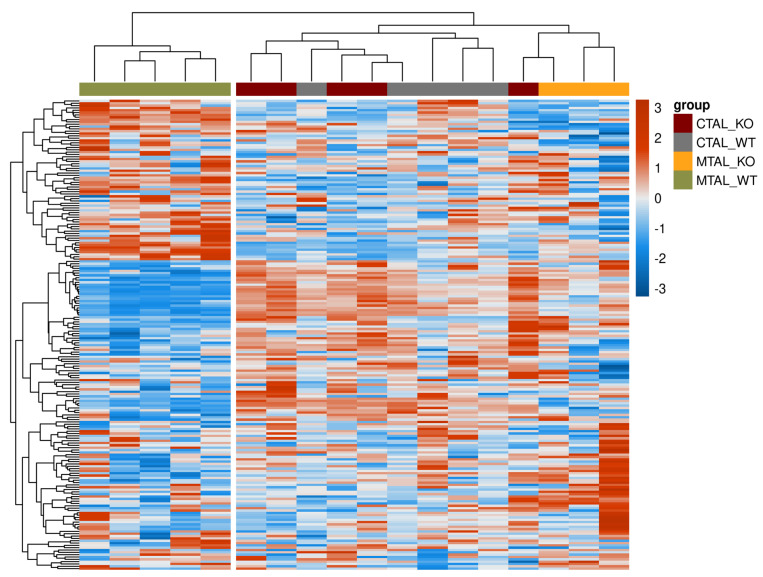
Supervised clustering of cKO MTAL, WT MTAL, cKO CTAL and WT CTAL based on a list of genes with a likely preferential expression in the TAL. The heatmap was generated using pheatmap R package on five pools of WT CTAL, five pools of cKO CTAL, five pools of WT MTAL and three pools of cKO MTAL. Clustering was constructed on 202 genes using correlation methods and complete linkage. The gradient of colors represents the expression level (z-score).

The analysis of gene expression patterns in all replicates showed that WT MTAL did not cluster with the other replicates; no hierarchical clustering could be found between WT CTAL, cKO CTAL and cKO MTAL (Figure 8). The GSEA analysis of the custom list of 222 genes with a likely preferential expression in the TAL also distinguished WT MTAL from the other three groups; no hierarchical clustering could be found between WT CTAL, cKO CTAL and cKO MTAL (Figure 9). The expression of phosphatases (Figure S14) and kinases (Figure S15) allowed a clustering of most WT MTAL apart from cKO MTAL, cKO CTAL and WT CTAL. The gene expression of transcription factors also isolated most WT MTAL from the other samples (Figure S16). *Cldn16*, *Pth1r*, *Casr* and *Vdr* expression was lower in WT MTAL than in any other group (Figure 1 and Figure 4).

## 3. Discussion

TAL is commonly considered a whole, despite a number of anatomical and functional differences between its medullary and cortical parts. We took advantage of manual dissection to separately study MTAL and CTAL and to eliminate nearby tubular segments. We profiled gene expression along MTAL and CTAL to determine the effect of *Cldn10* deletion on the transcriptomes of each specific tubular segment.

We confirmed the difference in gene expression pattern between WT MTAL and WT CTAL, with a higher expression of *Cldn10* [24,25] and a lower expression of *Cldn16* [24,25], *Clcnkb* [24,25], *Kcnj16* [24,25], *Casr* [24,25,32], *Pth1r* [24,25] and *Vdr* [24,25] in WT MTAL than in WT CTAL. Extending our analyzes to phosphatases, kinases and transcription factors (including *Foxq1* and *Irx3*), we also showed differences between MTAL and CTAL; specifically, we confirmed the lower expression of *Foxq1* and of Iroquois homeobox transcription factor *Irx3* in MTAL than in CTAL [24,25].

One notable result is that depletion in *Cldn10* has more effects on MTAL than on CTAL transcriptome; in MTAL, it alters the expression of more than 600 genes, including several with an important role in the control of transepithelial ion transport such as *Pth1r*, *Casr* and *Vdr* [23,33,34,35]. In the CTAL, *Cldn10* depletion causes a significant change in the expression of only 76 genes; here, it altered slightly, albeit significantly, the expression of *Pth1r*, *Casr* and *Vdr*. Overall, the transcriptome of cKO MTAL is closer to that of CTAL than to that of MTAL, suggesting that the expression of *Cldn10* is important not only for the properties of the paracellular pathway but also for the overall properties of the tubular segment where it is expressed.

CLDN10 is expressed at all tight junction in the ISOM TAL and in a significant percentage of tight junctions in the CTAL [29,30]; here, it is important for normal NaCl reabsorption by these tubular segments, as shown by the phenotype of cKO mice as well as that of patients with HELIX syndrome who exhibit a renal loss of NaCl with secondary hyperaldosteronism [4,5,6,7,8,9,10]. In actual fact, HELIX syndrome is the only syndrome with NaCl loss stemming from the TAL which is due to a defect in paracellular transport, underlining the importance of paracellular ion transport in this segment (and others) in homeostasis. The heterogeneity of expression of CLDN10 is an illustration of the overall axial and transverse heterogeneity of the TAL [1,2]. In the ISOM TAL and CTAL, tight junctions that do not express CLDN10 express CLDN16 and CLDN19, which increases their permeability to Ca^2+^ and Mg^2+^ [10,11,16]. The simplest hypothesis to explain this observation is based on the mutual exclusion of CLDN10, on the one hand, and CLDN16 and 19, on the other hand, within a given tight junction. In reality, CLDN10 is only able to achieve cis- and trans-interactions with itself [29]. However, the results of the present study suggest that CLDN10 has more complex effects on the TAL than just changing the composition of the tight junction. The depletion in *Cldn10* slightly increases the expression of *Cldn16* and 19 in both ISOM TAL and CTAL. When needed (for example, under conditions of low calcium and magnesium intake), an increase in paracellular permeability to Ca^2+^ and Mg^2+^ is an appropriate adaptive process; this can be obtained by increasing the paracellular permeability to divalent cations in tight junctions expressing CLDN16-CLDN19 and/or by a “replacement” of CLDN10 tight junctions by CLDN16-CLDN19 tight junctions in MTAL and/or CTAL. Therefore, we hypothesize that the plasticity of tight junctions is necessary to maintain homeostasis. Yet, the effect of the Cldn10 depletion is not limited to tight junction proteins expression and/or function. Cldn10 depletion is associated with an increase in the expression of *Cldn14*, *Pth1r*, *Casr* and *Vdr* genes in both segments, which all encode proteins that are important for the control of divalent cation reabsorption [23,32,33,34,35,36,37]. We know that the paracellular permeability to Ca^2+^ is positively regulated by parathyroid hormone and negatively regulated by the CaSR expressed at the basolateral membrane of the TAL [23,32]: the inhibition of CaSR increased tubular Ca^2+^ reabsorption [32]. Furthermore, the depletion in *Cldn10* changes the expression of several genes encoding proteins involved in NaCl reabsorption, such as *Clcnkb*, *Kcnj1* and *Kcnj10*; it affects the expression of *Umod* as well as that of several kinases, phosphatases and transcription factors.

Chen, L., et al. reported by single-cell transcriptome analysis two CTAL cell clusters, one expressing a high level of *Cldn10* and *Ptger3* and the other one expressing a high level of *Cldn16*, *Foxq1* and *Irx3* [28]. One could hypothesize that a depletion in *Cldn10* merely leads to the replacement of *Cldn10* expressing cells by a similar number of *Cldn16* expressing cells; however, in that case, some genes that are enriched in Cldn10-expressing cells, such as *Ptger3*, should be decreased in cKO CTAL or MTAL, which is not the case. Rather, the results of the present study suggest that depletion in *Cldn10* changes a significant proportion but not the totality of the MTAL and CTAL transcriptomes.

The interpretation of the data was limited by the use of a conditional knock-out model, in which the *Cldn10* deletion was not complete. However, this was the only mouse model with a TAL-specific *Cldn10* gene defect as the global knock-out of *Cldn10* in mice is lethal. We made sure to retain only the cKO TAL pools with significantly lower *Cldn10* expression than in WT TAL for analysis, allowing us to draw conclusions.

The importance of the functional properties of tight junction has long been overlooked. Our study highlights the potentially larger role of tight junction proteins in cellular phenotype and gene expression pattern. We demonstrate that the expression of *Cldn10* encoding a tight junction protein is associated with the gene expression of many ion channels, phosphatases and kinases. This kind of study should be performed in other epithelia such as in the intestine where the expression of CLDN varies along both the crypt–lumen axis and the longitudinal axis of the intestinal tract [38,39]. Thus, we could identify whether our findings regarding the reprogramming of gene expression by a claudin are shared by other tissues.

## 4. Materials and Methods

### 4.1. Animals

Mice that were conditionally *Cldn10*-deficient due to Ksp-cadherin-driven Cre-recombinase expression in the distal nephron (*Cldn10*^fl/fl^; Ksp-Cre, referred to as cKO) and their control littermates (*Cldn10*^fl/fl^, referred to as WT) were used [9].

All experiments were performed on adult, 4–10-month-old male mice (>90% C57BL/6 background). Before the experiments, mice were kept in standard cages at 21–22 °C, in a 12 h/12 h light/dark cycle, and were fed regular chow and had *ad libitum* access to drinking water. The experimental protocols were approved by the Comité d’éthique pour l’expérimentation animale Charles Darwin and the Ministere de la Recherche (Agreement #15518 2018061419183510).

### 4.2. Microdissection and RNA Sequencing

Pools of 150–200 thick ascending limbs of Henle’s loop were isolated from the inner stripe of outer medulla and the cortex (MTAL and CTAL, respectively (150 mm)) under binocular loupes, according to well-defined morphologic characteristics, from a liberase-treated kidney, as previously described [40]. Briefly, the left kidney was perfused in situ via the abdominal aorta with 3 mL of dissection solution (modified Hank’s solution [40]) containing 0.012% liberase (Blenzymze2, Roche Diagnostics, Meylan, France). The kidney was cut in thin pyramids and incubated in a dissection solution containing 0.005% liberase for 25 min at 30 °C. Each pool of MTAL or CTAL was dissected from a distinct animal.

After microdissection, pools of tubules were immediately transferred to RLT buffer (RNeasy micro-kit, Quiagen, Les Ulis, France). RNA was then extracted according to the manufacturer’s protocol.

### 4.3. Whole-Transcriptome RNA Sequencing

The RNA integrity (RNA Integrity Score ≥ 7.0) was checked using the Agilent FragmentAnalyzer (Agilent, Les Ulis, France) and the quantity was determined using Qubit (Invitrogen, Villebon-sur-Yvette, France). A SureSelect Automated Strand Specific RNA Library Preparation Kit was used according to the manufacturer’s instructions with the Bravo Platform. Briefly, 50 ng of total RNA per sample was used for poly-A mRNA selection using oligo(dT) beads and was subjected to thermal mRNA fragmentation. The fragmented mRNA samples were subjected to cDNA synthesis and were further converted into double stranded DNA using the reagents supplied in the kit, and the resulting dsDNA was used for library preparation. The final libraries were bar-coded, purified, pooled together in equal concentrations and subjected to paired-end sequencing on a Novaseq-6000 sequencer (Illumina, San Diego, CA, USA) at Gustave Roussy (Villejuif, France).

### 4.4. RNA-Seq Analyses

The quality of raw data was evaluated with FastQC [41]. Poor quality sequences and adapters were trimmed or removed with the fastp tool, with default parameters, to retain only good quality paired reads [42]. Illumina DRAGEN bio-IT Platform (v3.10.11) was used for mapping on the mm10 reference genome and quantification with the gencode v25 annotation gtf file. Library orientation, composition and coverage along transcripts were checked with Picard tools.

After quality controls, 2 pools of cKO MTAL were excluded. One pool of cKO CTAL was excluded due to Cldn10 expression level being similar to that of WT CTAL.

The following analyses and data visualizations were conducted with an interactive R Shiny software named Quby (https://dac.institutducerveau-icm.org (accessed from October 2023 to January 2024)). The count matrix was filtrated at a CPM threshold ≥ 3 on at least 5 samples, except for comparisons involving cKO MTAL which were filtrated at a CPM threshold ≥ 3 on at least 3 samples. Data were normalized with an edgeR (v3.28.0) Bioconductor package [43], prior to differential analysis with a glm framework likelihood ratio test from DESeq2 (v.1.34) workflow [44]. Multiple hypothesis-adjusted p-values were calculated with the Benjamini–Hochberg procedure to control FDR. Differential analysis results were analyzed with a log2 foldchange threshold of 1 and a FDR of <0.05.

Finally, enrichment analysis was conducted with clusterProfiler R package (v4.2.2) [45] with a gene set enrichment analysis, with 4 personalized gene lists: the first encompassed 222 genes likely to be expressed in TAL (Table S2), the second comprised 213 genes that encoded phosphatases (Table S6), the third encompassed 478 genes that encoded kinases (Table S7) and the last one comprised 121 genes that encoded transcription factors (Table S8).

### 4.5. Other Data Analysis

The expression of specific genes is represented as dots corresponding to the individual cpm value of each WT or cKO MTAL or CTAL pool and expressed as median and interquartile range. Groups were compared using Mann–Whitney U test. *p* < 0.05 was regarded as statistically significant. Statistical analyses were performed using Prism 10 software.

## Data Availability

Raw sequence reads and from RNA-seq and processed data are available from GEO under accession number GSE252783.

## Supplementary Materials

The following supporting information can be downloaded at: https://www.mdpi.com/article/10.3390/ijms25074008/s1.
