# Supplementary material for: Claudin-10 Expression and the Gene Expression Pattern of Thick Ascending Limb Cells
Source: Int J Mol Sci. 2024 Apr 3;25(7):4008. doi: 10.3390/ijms25074008 (PMC11011785; doi:10.3390/ijms25074008)
Supplement: Supplementary file 1 [file ijms-25-04008-s001.zip › ijms-2877292-Supplementary Materials.pdf]

Supplementary Materials

Figure S1: Gene expression analyses of *Cldn10* (A), *Slc12a1* (encoding the Na^+-K^+-$2Cl^-$ cotransporter NKCC2) (B), *Slc2a2* (encoding the glucose transporter 2) (C), *Slc12a3* (encoding the thiazide sensitive Na^+-Cl^- cotransporter NCC) (D) and *Aqp2* (Aquaporin-2) (E) in WT and cKO CTAL and MTAL. Medians and interquartile ranges are shown (Mann-Whitney test); Counts per millions (cpm).

Figure S2: Heatmap showing gene expression pattern for all replicates of cKO MTAL and WT MTAL. The heatmap was generated using pheatmap R package on 3 pools of cKO MTAL and 5 pools of WT MTAL. Clustering was constructed on genes using Euclidean methods and complete linkage. The gradient of colors represents the expression level (z-score). More expressed and less expressed genes are reported in Table S1.

Figure S3: Heatmap for gene expression of phosphatases in cKO MTAL and WT MTAL. The heatmap was generated using pheatmap R package on 3 pools of cKO MTAL and 5 pools of WT MTAL. Clustering was constructed on 212 genes using Euclidean methods and complete linkage. The gradient of colors represents the expression level (z-score).

Figure S4: Heatmap for gene expression of kinases in cKO MTAL and WT MTAL. The heatmap was generated using pheatmap R package on 3 pools of cKO MTAL and 5 pools of WT MTAL. Clustering was constructed on 476 genes using Euclidean methods and complete linkage. The gradient of colors represents the expression level (z-score).

Figure S5: Heatmap for gene expression of transcription factors in cKO MTAL and and WT MTAL.

The heatmap was generated using pheatmap R package on 3 pools of cKO MTAL and 5 pools of WT MTAL. Clustering was constructed on 120 genes using Euclidean methods and complete linkage. The gradient of colors represents the expression level (z-score).

Figure S6: Heatmap showing gene expression pattern for all replicates of cKO CTAL and WT CTAL. The heatmap was generated using pheatmap R package on 5 pools of cKO CTAL and 5 pools of WT CTAL. Clustering was constructed on genes using Euclidean methods and complete linkage. The gradient of colors represents the expression level (z-score). More expressed and less expressed genes are reported in Table S3.

Figure S7: Heatmap showing gene expression pattern for all replicates of WT MTAL and WT CTAL. The heatmap was generated using pheatmap R package on 5 pools of WT CTAL and 5 pools of WT MTAL. Clustering was constructed on genes using Euclidean methods and complete linkage. The gradient of colors represents the expression level (z-score). More expressed and less expressed genes are reported in Table S4.

Figure S8: Heatmap for gene expression of phosphatases in WT MTAL and WT CTAL. The heatmap was generated using pheatmap R package on 5 pools of WT CTAL and 5 pools of WT MTAL. Clustering was constructed on 212 genes using Euclidean methods and complete linkage. The gradient of colors represents the expression level (z-score).

Figure S9: Heatmap for gene expression of kinases in WT MTAL and WT CTAL. The heatmap was generated using pheatmap R package on 5 pools of WT CTAL and 5 pools of WT MTAL. Clustering was constructed on 477 genes using Euclidean methods and complete linkage. The gradient of colors represents the expression level (z-score).

Figure S10: Heatmap for gene expression of transcription factors in WT MTAL and WT CTAL.

The heatmap was generated using pheatmap R package on 5 pools of WT CTAL and 5 pools of WT MTAL. Clustering was constructed on 119 genes using Euclidean methods and complete linkage. The gradient of colors represents the expression level (z-score).

Figure S11: Heatmap showing gene expression pattern for all replicates of cKO MTAL and cKO CTAL. The heatmap was generated using pheatmap R package on 5 pools of cKO CTAL and 3 pools of cKO MTAL. Clustering was constructed on genes using Euclidean methods and complete linkage. The gradient of colors represents the expression level (z score). More expressed and less expressed genes are reported in Table S5.

Figure S12: Heatmap for gene expression of phosphatases in cKO MTAL and cKO CTAL.
The heatmap was generated using pheatmap R package on 5 pools of cKO CTAL and 3 pools of cKO MTAL. Clustering was constructed on 211 genes using Euclidean methods and complete linkage. The gradient of colors represents the expression level (z-score).

Figure S13: Heatmap for gene expression of transcription factors in cKO MTAL and cKO CTAL.

The heatmap was generated using pheatmap R package on 5 pools of cKO CTAL and 3 pools of cKO MTAL. Clustering was constructed on 120 genes using Euclidean methods and complete linkage. The gradient of colors represents the expression level (z-score).

Figure S14: Heatmap for gene expression of phosphatases in cKO MTAL, WT MTAL, cKO CTAL and WT CTAL The heatmap was generated using pheatmap R package on 5 pools of WT CTAL, 5 pools of cKO CTAL, 5 pools of WT MTAL and 3 pools of cKO MTAL. Clustering was constructed on 191 genes using correlation methods and complete linkage. The gradient of colors represents the expression level (z-score).

Figure S15: Heatmap for gene expression of kinases in cKO MTAL, WT MTAL, cKO CTAL and WT CTAL The heatmap was generated using pheatmap R package on 5 pools of WT CTAL, 5 pools of cKO CTAL, 5 pools of WT MTAL and 3 pools of cKO MTAL. Clustering was constructed on 450 genes using correlation methods and complete linkage. The gradient of colors represents the expression level (z-score).

Figure S16: Heatmap for gene expression of transcription factors in cKO MTAL, WT MTAL, cKO CTAL and WT CTAL The heatmap was generated using pheatmap R package on 5 pools of WT CTAL, 5 pools of cKO CTAL, 5 pools of WT MTAL and 3 pools of cKO MTAL. Clustering was constructed on 113 genes using correlation methods and complete linkage. The gradient of colors represents the expression level (z-score).

Table S1: More expressed and less expressed genes in cKO MTAL compared to WT MTAL. Three pools of cKO MTAL and 5 pools of WT MTAL were used for analysis. 637 genes were differentially expressed between WT MTAL and cKO MTAL replicates: 179 genes were less expressed and 458 genes more expressed in cKO MTAL compared to WT MTAL. Transcripts per million (TPM) are reported. Log2 Fold Change (logFC); Fold Change (FC); p-value (PValue); adjusted p-value (FDR)

Table S2: List of 222 genes with a likely preferential expression in the TAL

Table S3: More expressed and less expressed genes in cKO CTAL compared to WT CTAL. Five pools of cKO CTAL and 5 pools of WT CTAL were used for analysis. 66 genes were differentially expressed between cKO CTAL and WT CTAL: 29 genes were less expressed and 37 genes were more expressed in cKO CTAL compared to WT CTAL. Transcripts per million (TPM) are reported. Log2 Fold Change (logFC); Fold Change (FC); p-value (PValue); adjusted p-value (FDR).

Table S4: More expressed and less expressed genes in WT MTAL compared to WT CTAL. Five pools of WT MTALs and 5 pools of WT CTALs were used for analysis. 291 genes were differentially expressed between WT MTAL and WT CTAL: 205 genes were less expressed and 86 genes were more expressed in WT MTAL compared to WT CTAL. Transcripts per million (TPM) are reported. Log2 Fold Change (logFC); Fold Change (FC); raw p-value (PValue); adjusted p-value (FDR).

Table S5: More expressed and less expressed genes in cKO MTAL compared to cKO CTAL. Three pools of cKO MTAL and 5 pools of cKO CTAL were used for analysis. 359 genes were differentially expressed between cKO MTAL and cKO CTAL : 78 genes were less expressed and 281 genes were more expressed in cKO MTAL compared to cKO CTAL. Transcripts per million (TPM) are reported. Log2 Fold Change (logFC); Fold Change (FC); raw p-value (PValue); adjusted p-value (FDR).

Table S6: List of phosphatase genes

Table S7: List of kinase genes

Table S8: List of transcription factor genes
