# Supplementary figures and images for: Claudin-10 Expression and the Gene Expression Pattern of Thick Ascending Limb Cells
Source: Int J Mol Sci. 2024 Apr 3;25(7):4008. doi: 10.3390/ijms25074008 (PMC11011785; doi:10.3390/ijms25074008)

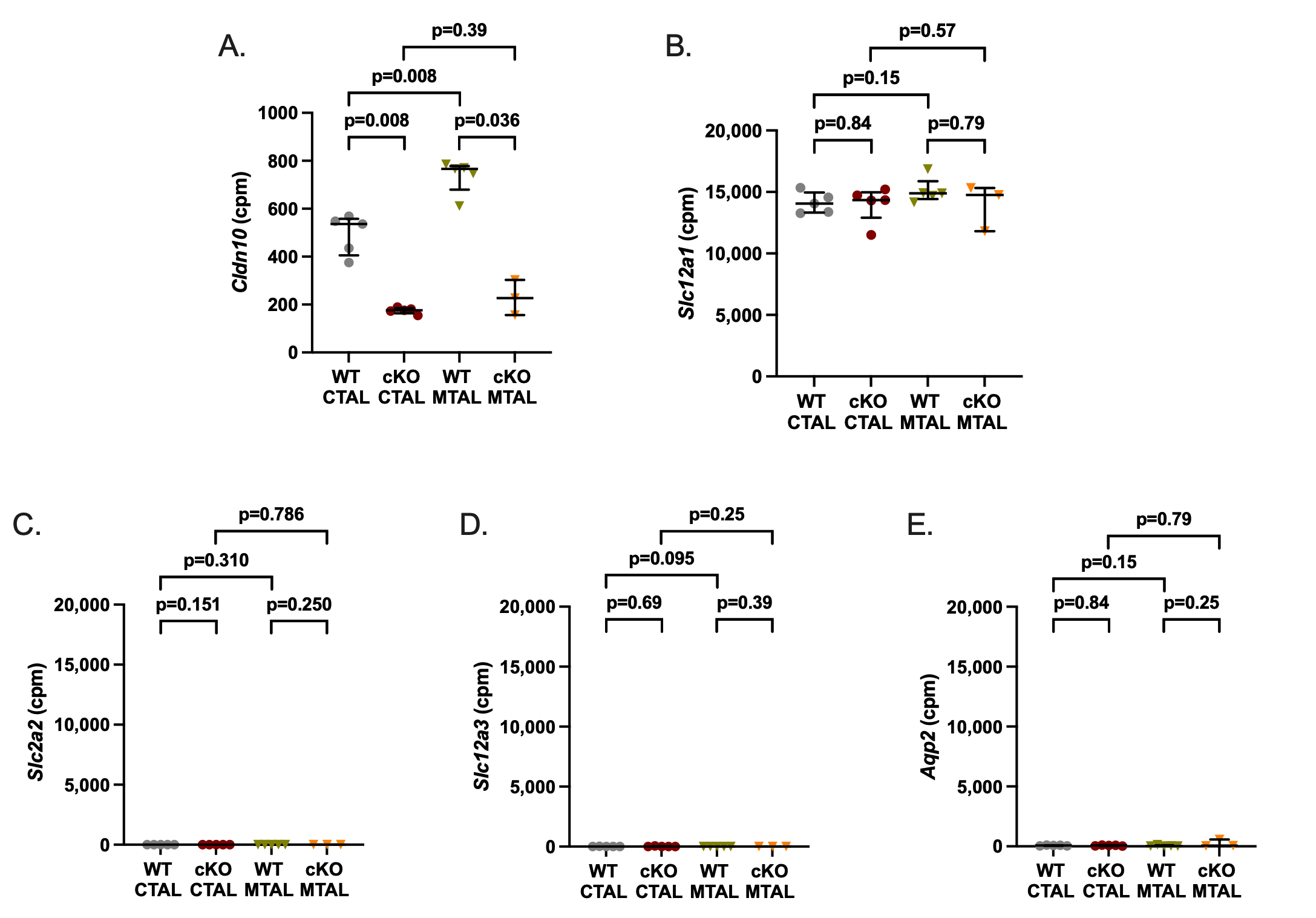

Supplement: Supplementary file 1 [file ijms-25-04008-s001.zip › Prot-Bertoye supplementary material/Supplemental Figure S1.tiff]

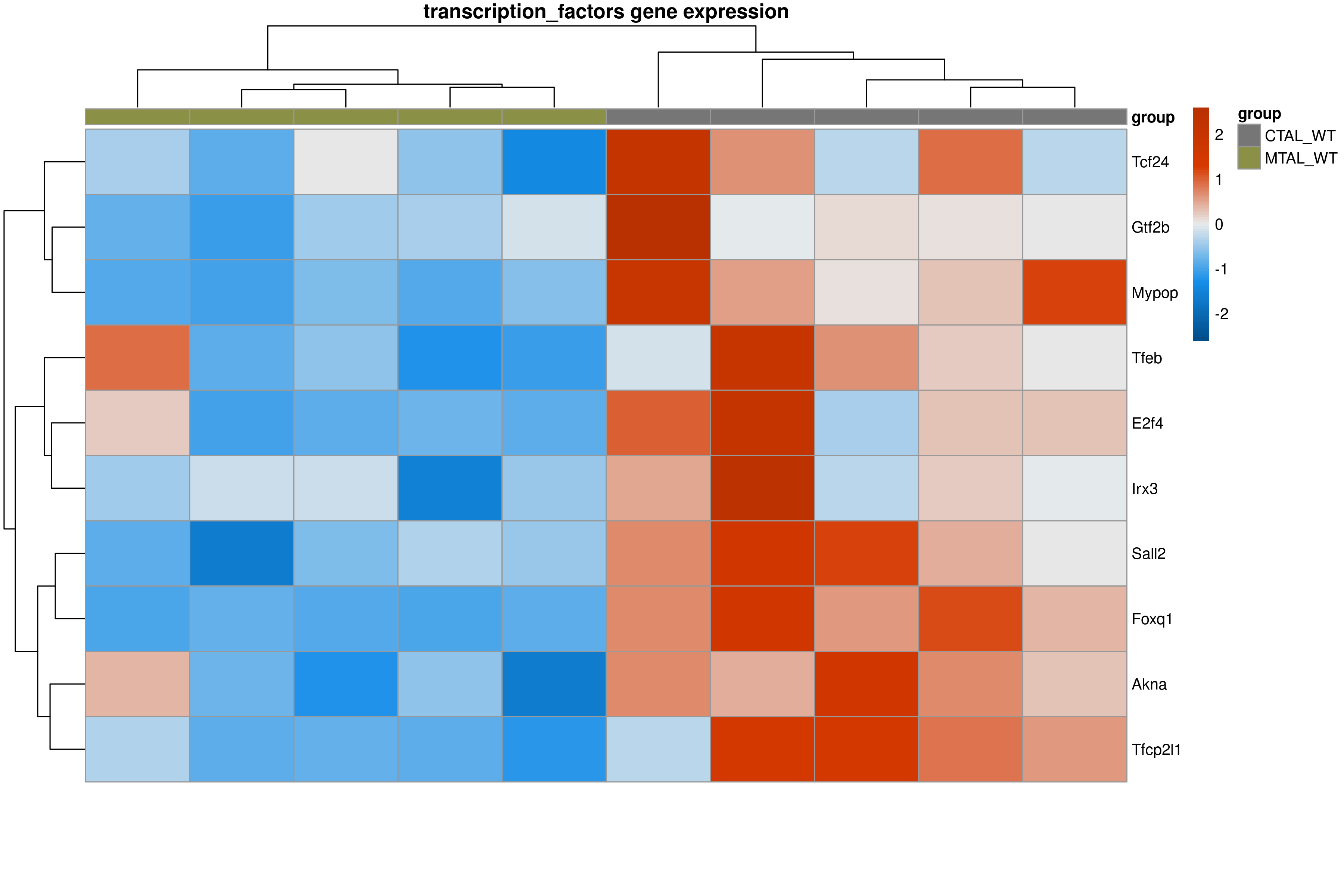

Supplement: Supplementary file 1 [file ijms-25-04008-s001.zip › Prot-Bertoye supplementary material/Supplemental Figure S10 WT MTAL vs WT CTAL transcription_factors.tiff]

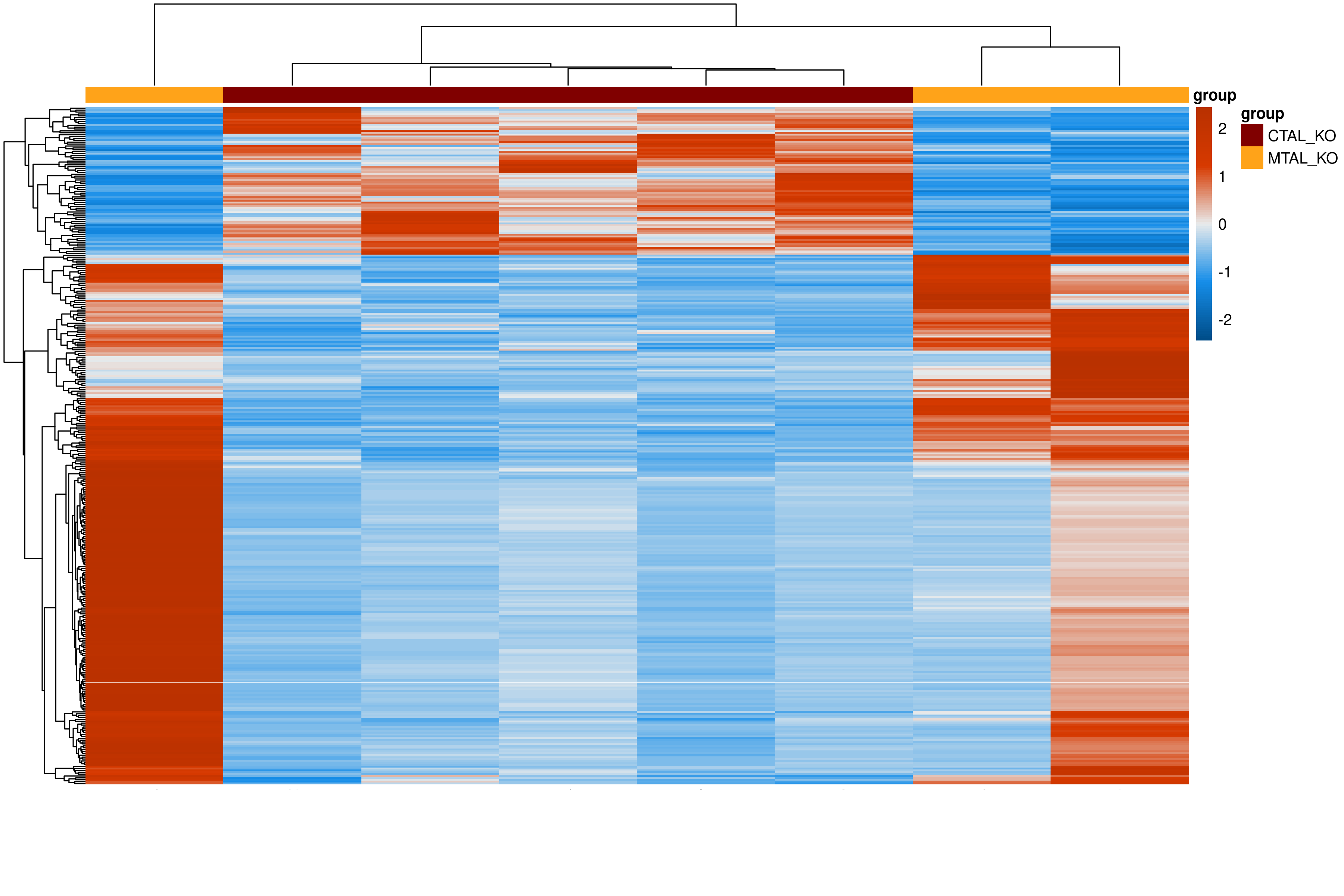

Supplement: Supplementary file 1 [file ijms-25-04008-s001.zip › Prot-Bertoye supplementary material/Supplemental Figure S11 cKO MTAL vs cKO CTAL global heatmap.tiff]

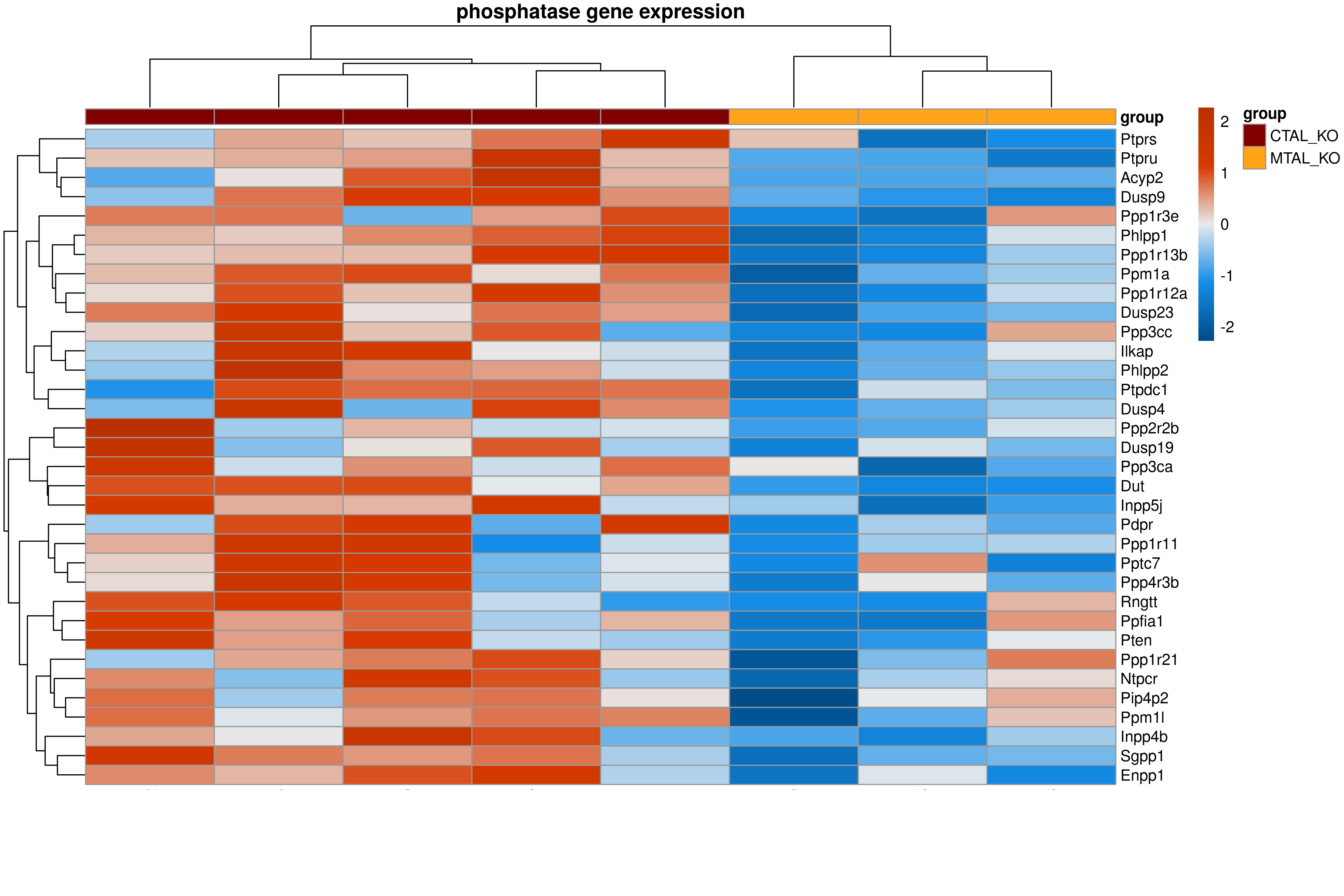

Supplement: Supplementary file 1 [file ijms-25-04008-s001.zip › Prot-Bertoye supplementary material/Supplemental Figure S12 cKO MTAL vs cKO CTAL phosphatase phosphatase.tiff]

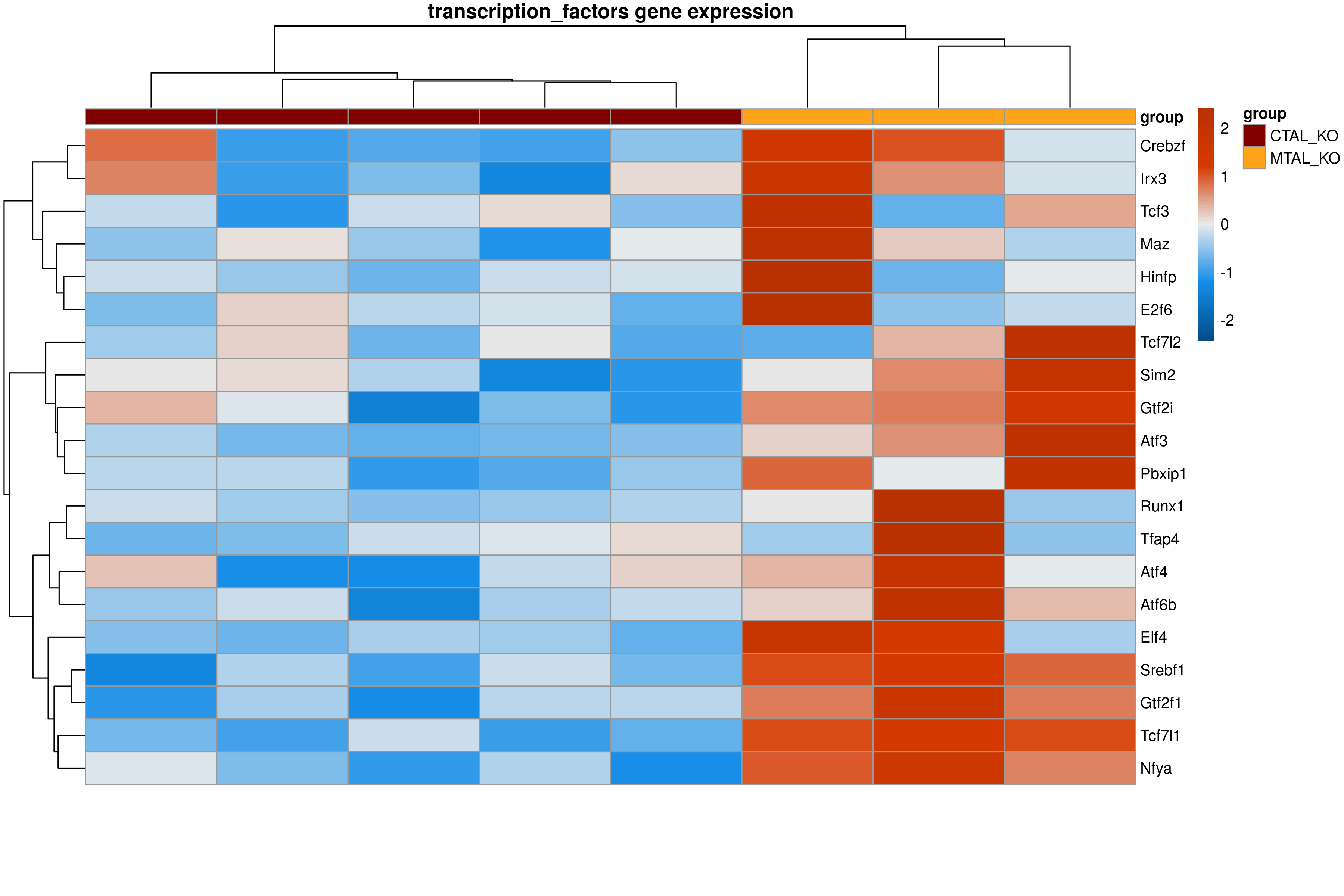

Supplement: Supplementary file 1 [file ijms-25-04008-s001.zip › Prot-Bertoye supplementary material/Supplemental Figure S13 cKO MTAL vs cKO CTAL transcription_factors.tiff]

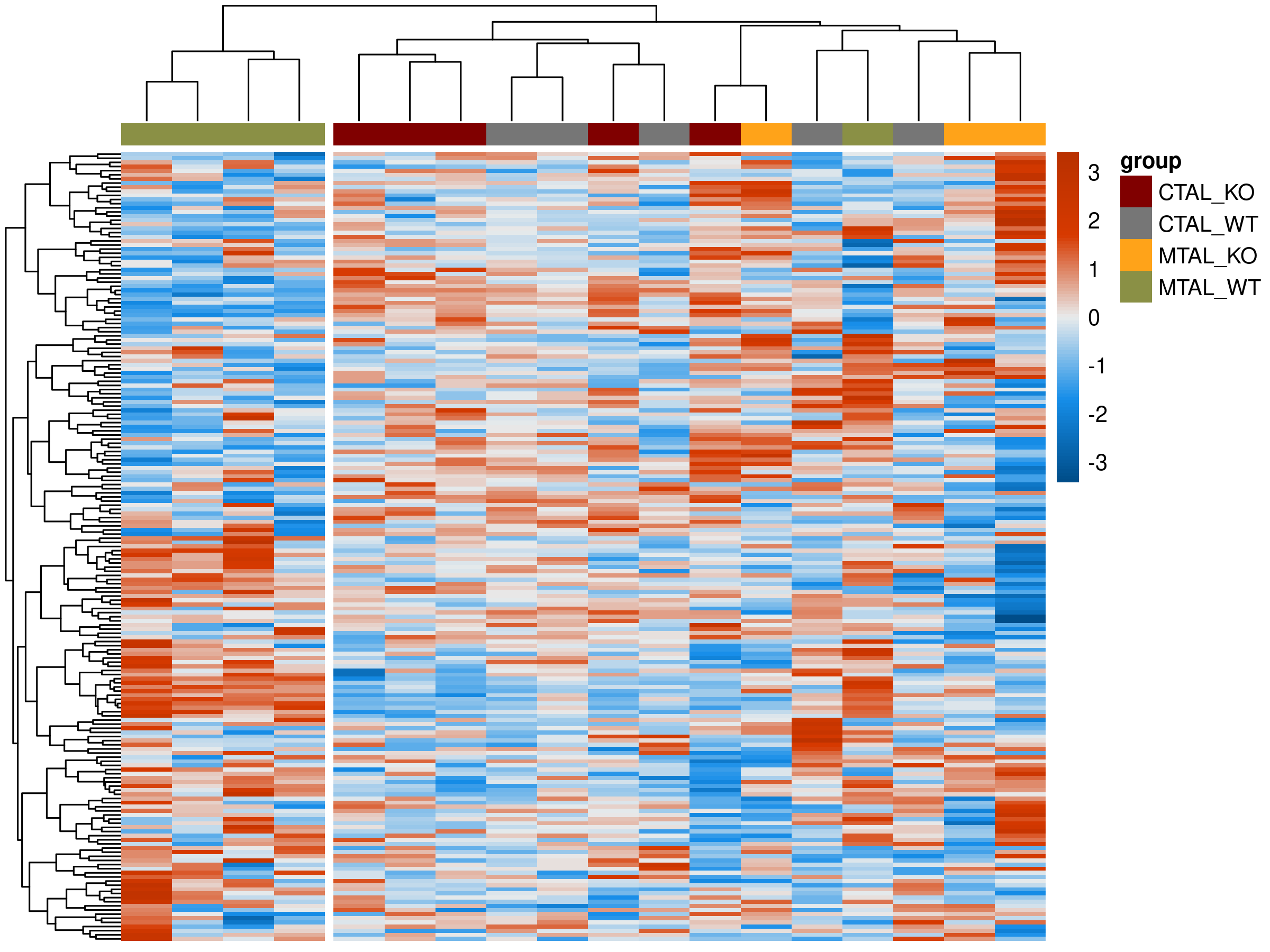

Supplement: Supplementary file 1 [file ijms-25-04008-s001.zip › Prot-Bertoye supplementary material/Supplemental Figure S14 Heatmap_phosphatase_genes.tiff]

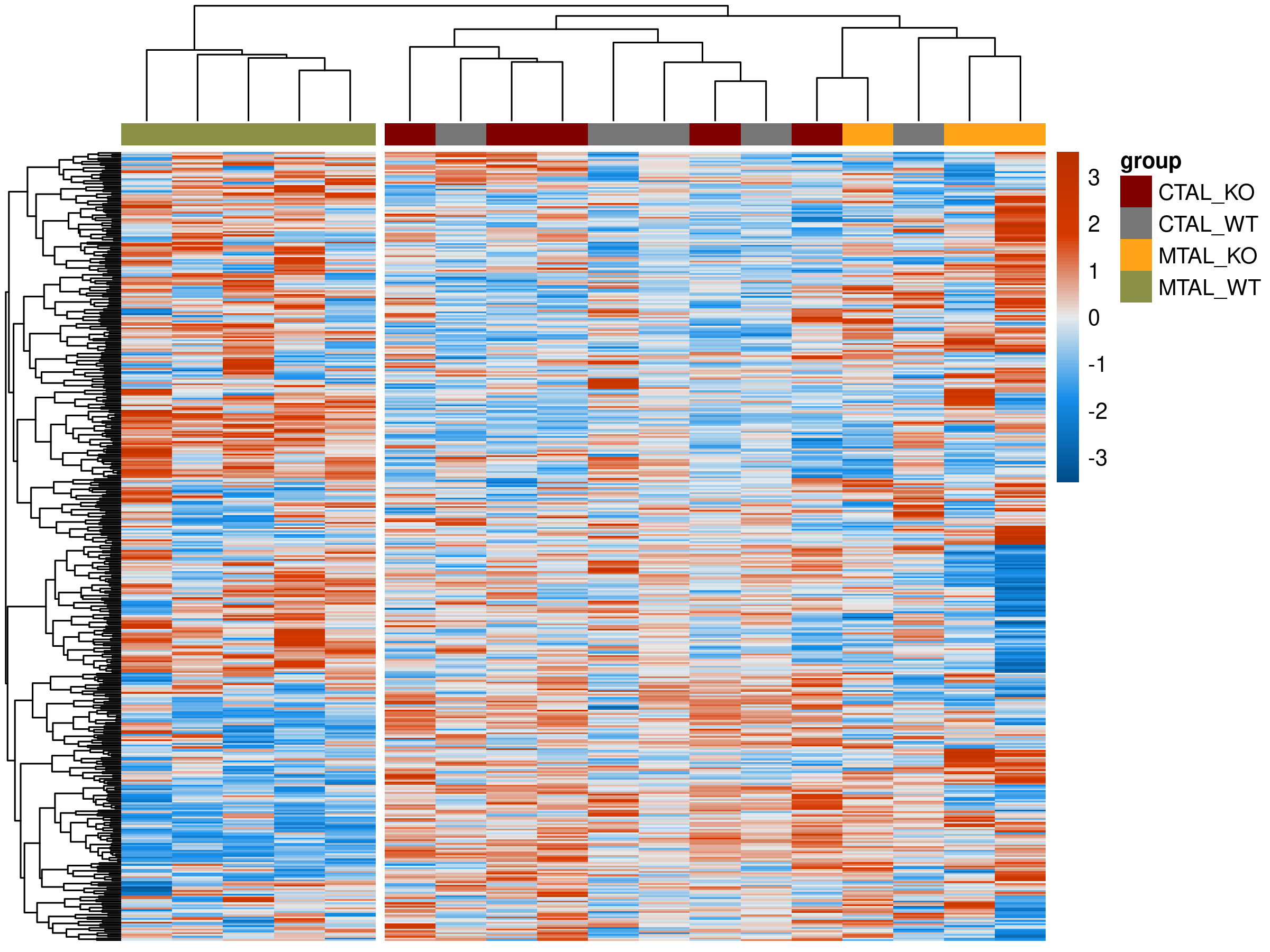

Supplement: Supplementary file 1 [file ijms-25-04008-s001.zip › Prot-Bertoye supplementary material/Supplemental Figure S15 Heatmap_kinase_genes.tiff]

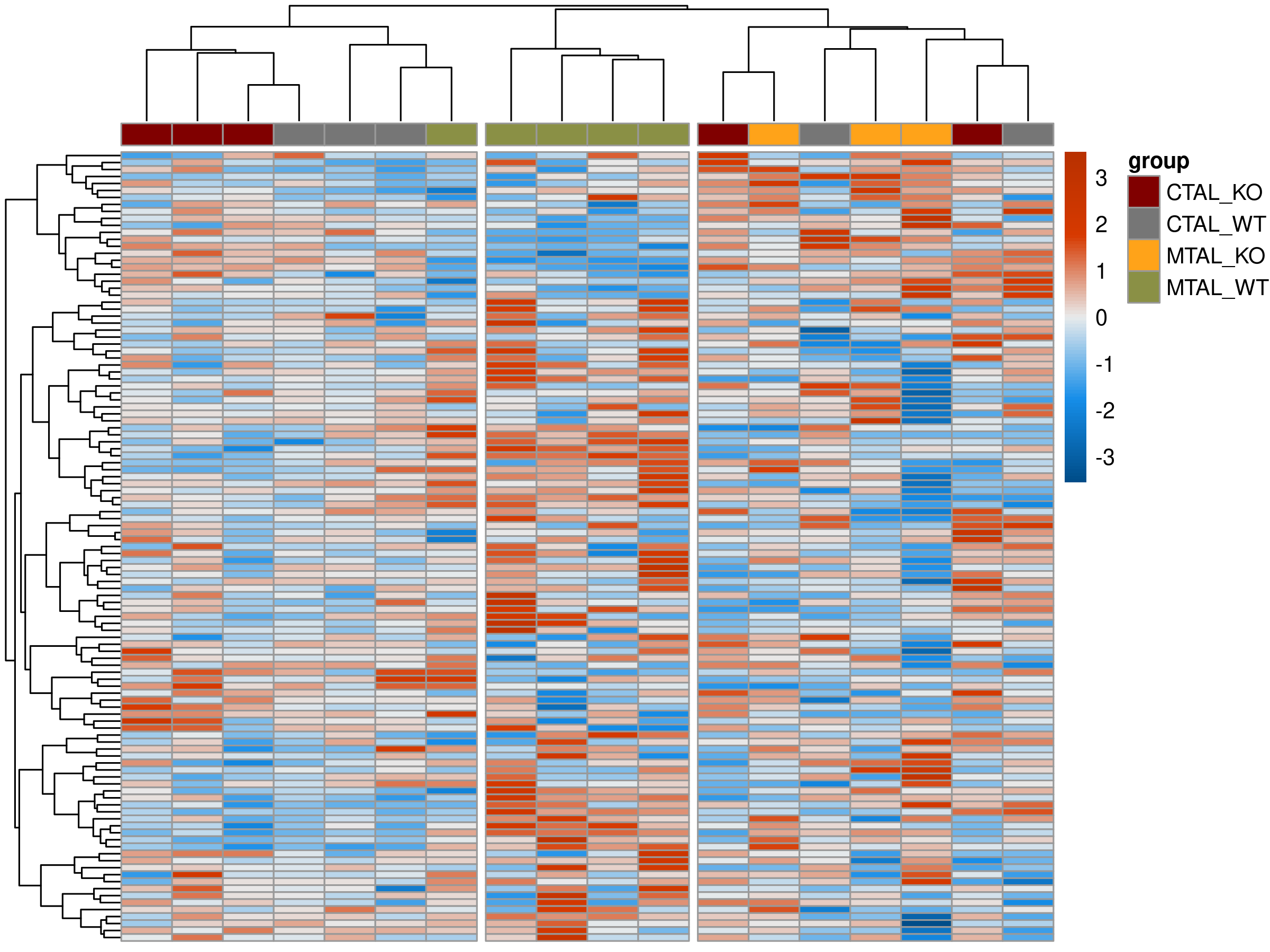

Supplement: Supplementary file 1 [file ijms-25-04008-s001.zip › Prot-Bertoye supplementary material/Supplemental Figure S16 Heatmap_transcription_factors_genes.tiff]

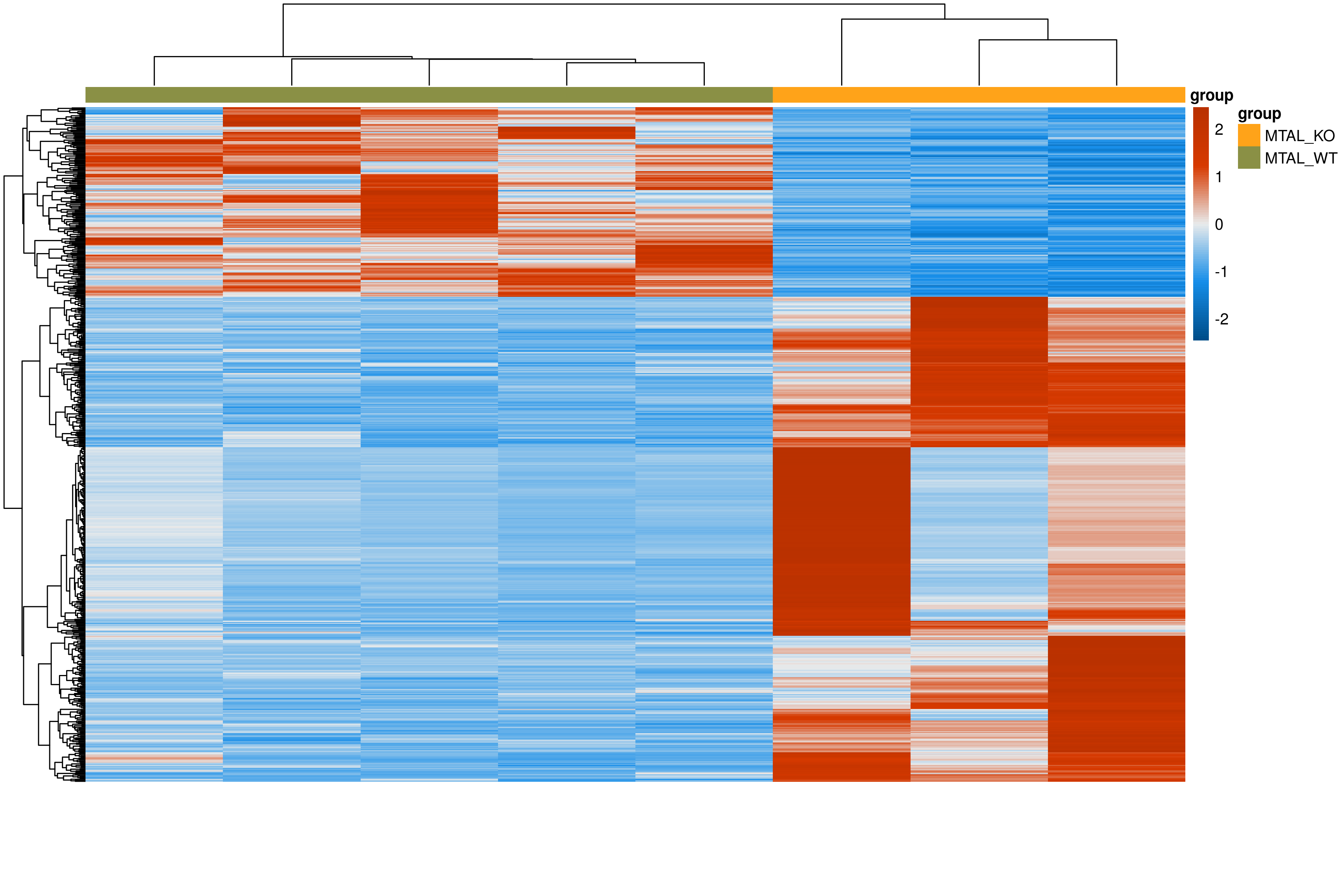

Supplement: Supplementary file 1 [file ijms-25-04008-s001.zip › Prot-Bertoye supplementary material/Supplemental Figure S2 cKO MTAL vs WT MTAL global-heatmap.tiff]

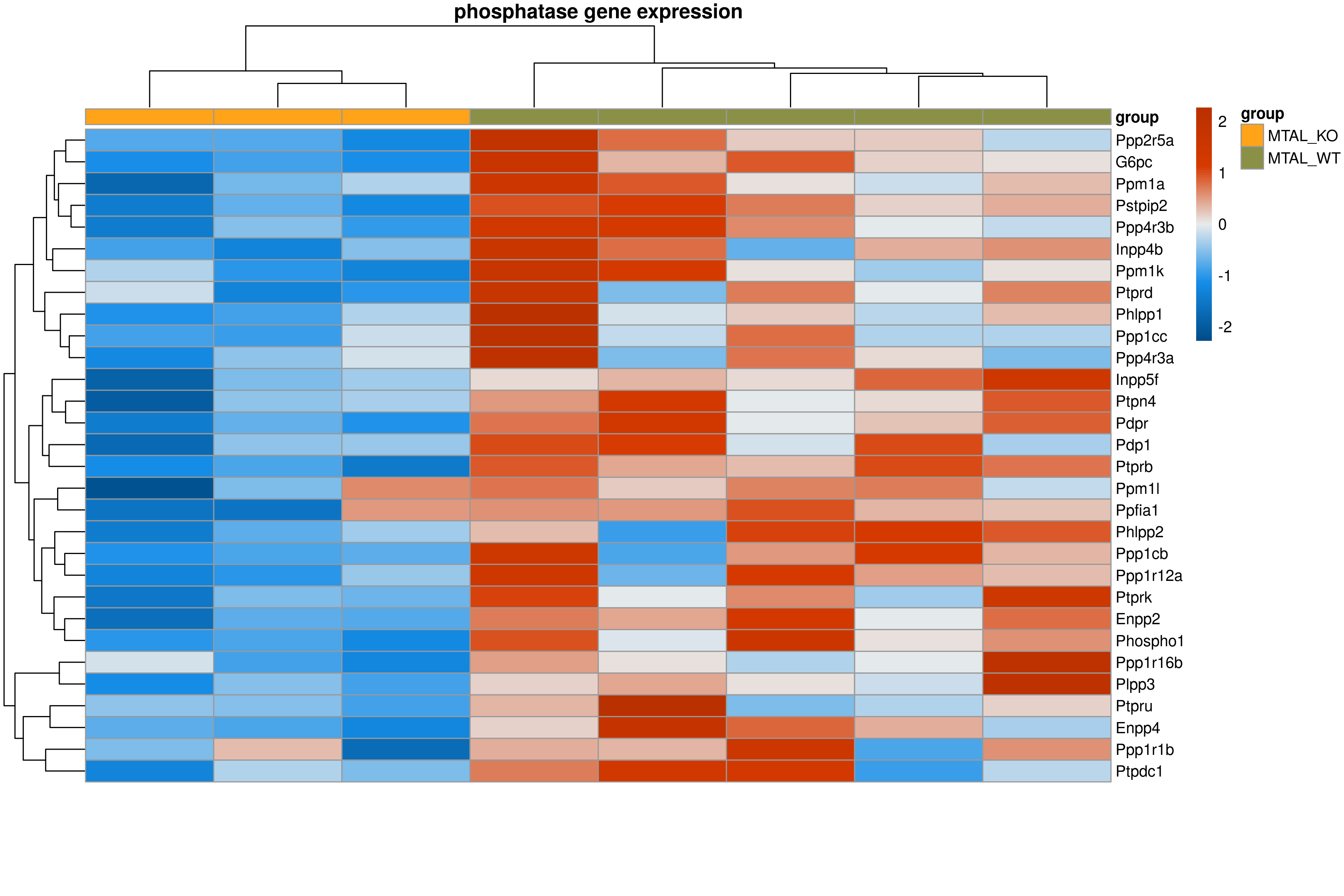

Supplement: Supplementary file 1 [file ijms-25-04008-s001.zip › Prot-Bertoye supplementary material/Supplemental Figure S3 cKO MTAL WT MTAL WT phosphatase.tiff]

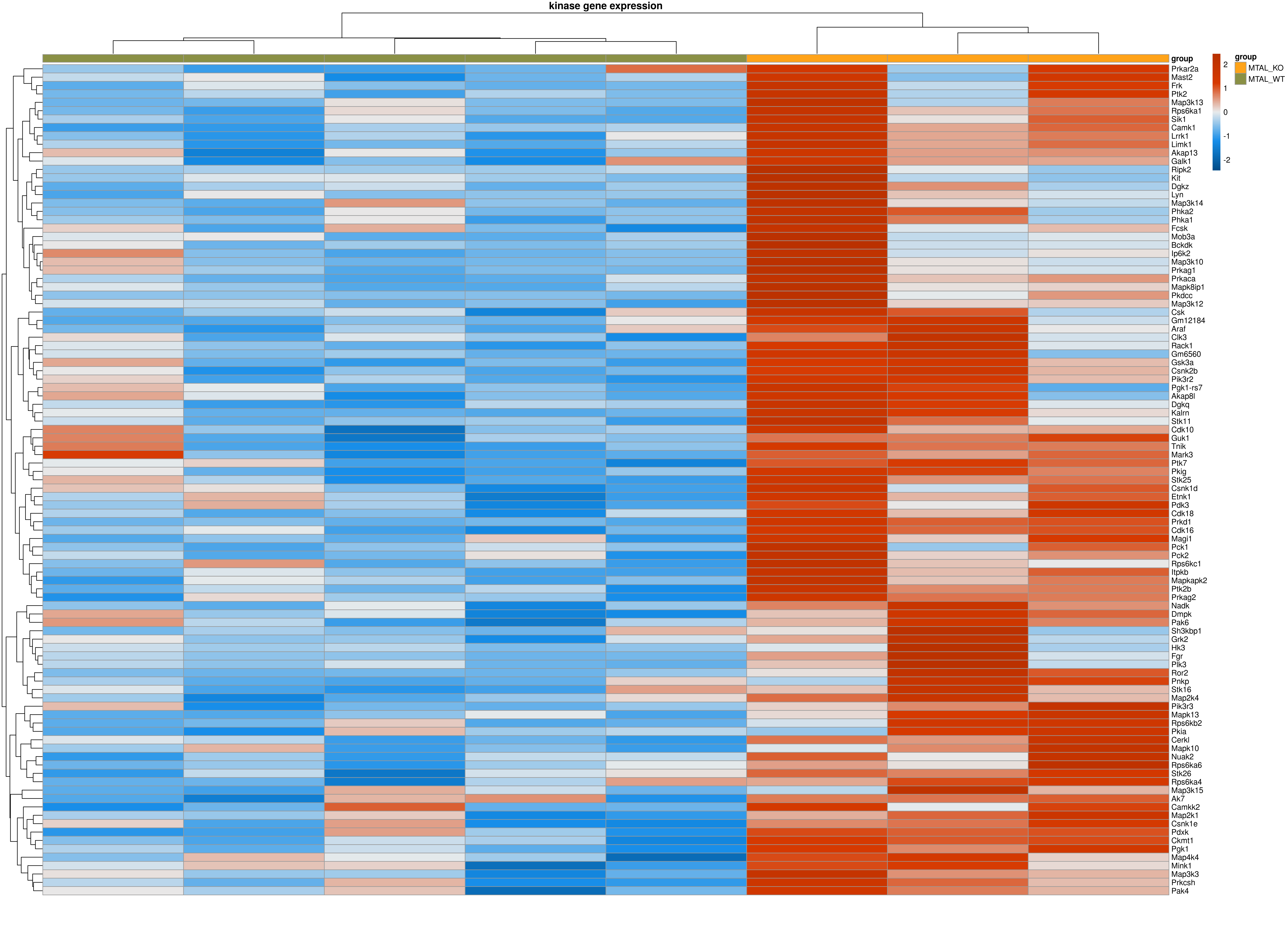

Supplement: Supplementary file 1 [file ijms-25-04008-s001.zip › Prot-Bertoye supplementary material/Supplemental Figure S4 cKO MTAL WT MTAL kinase.tiff]

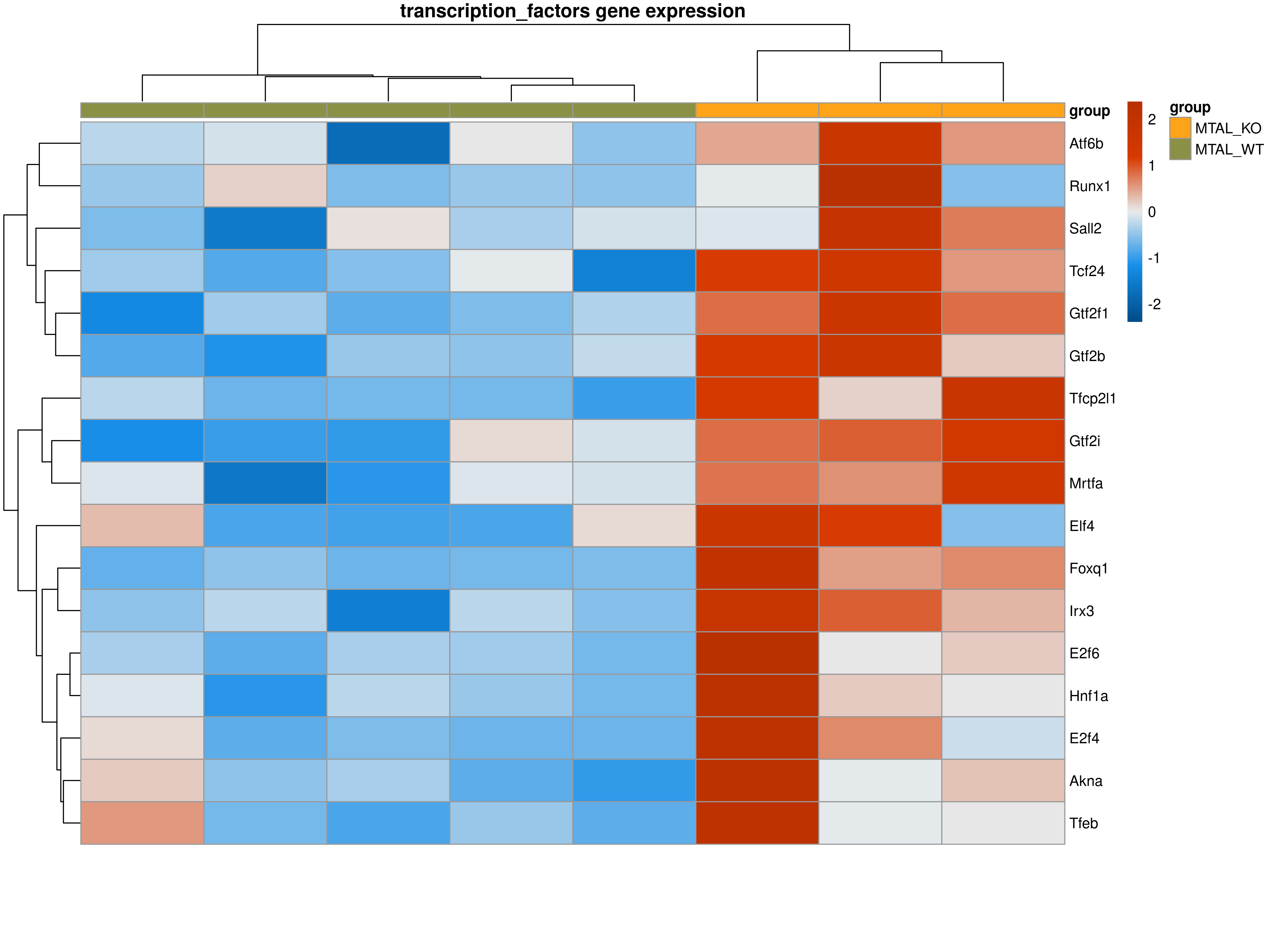

Supplement: Supplementary file 1 [file ijms-25-04008-s001.zip › Prot-Bertoye supplementary material/Supplemental Figure S5 cKO MTAL WT MTAL transcription_factors.tiff]

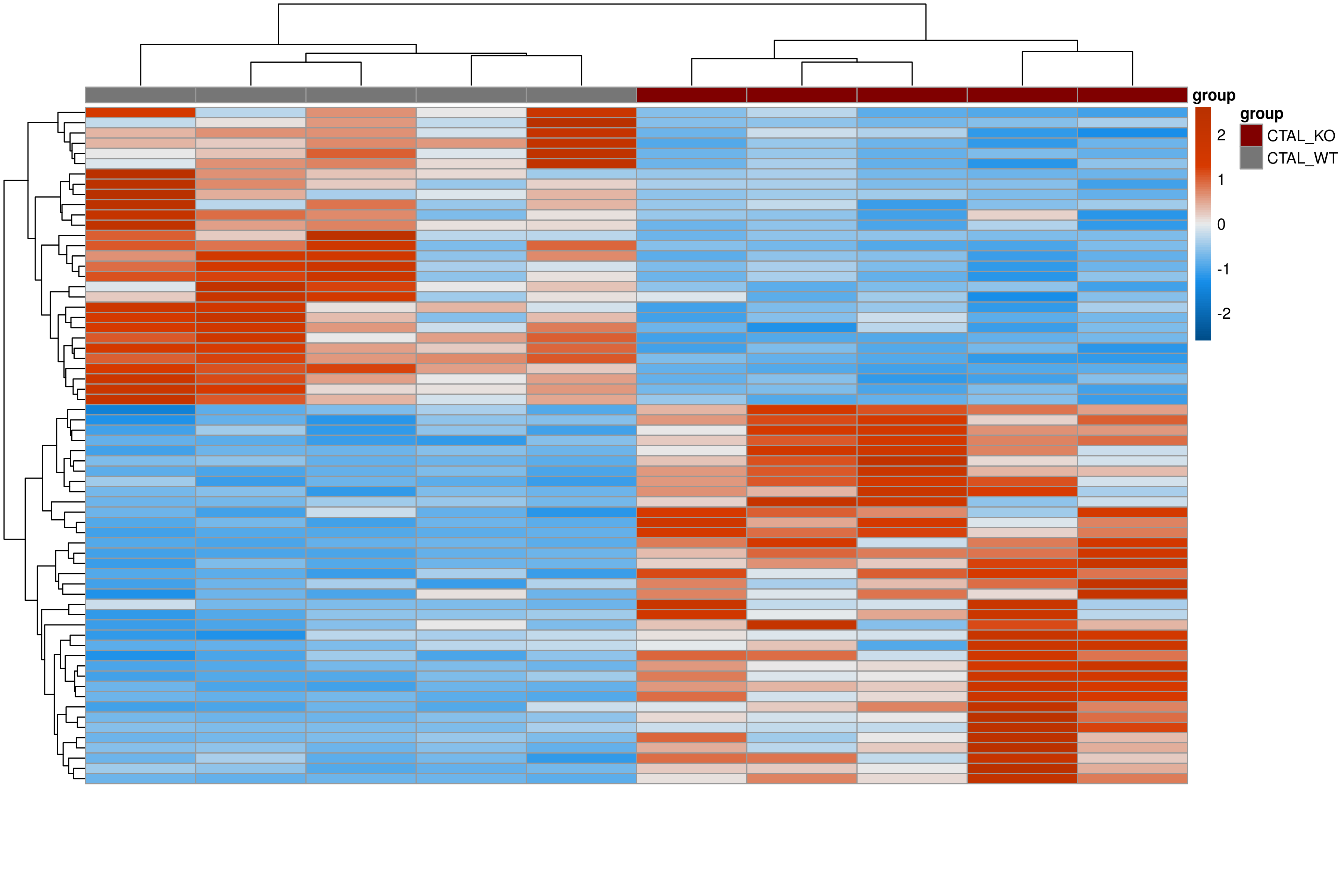

Supplement: Supplementary file 1 [file ijms-25-04008-s001.zip › Prot-Bertoye supplementary material/Supplemental Figure S6 cKO CTAL vs WT CTAL global heatmap.tiff]

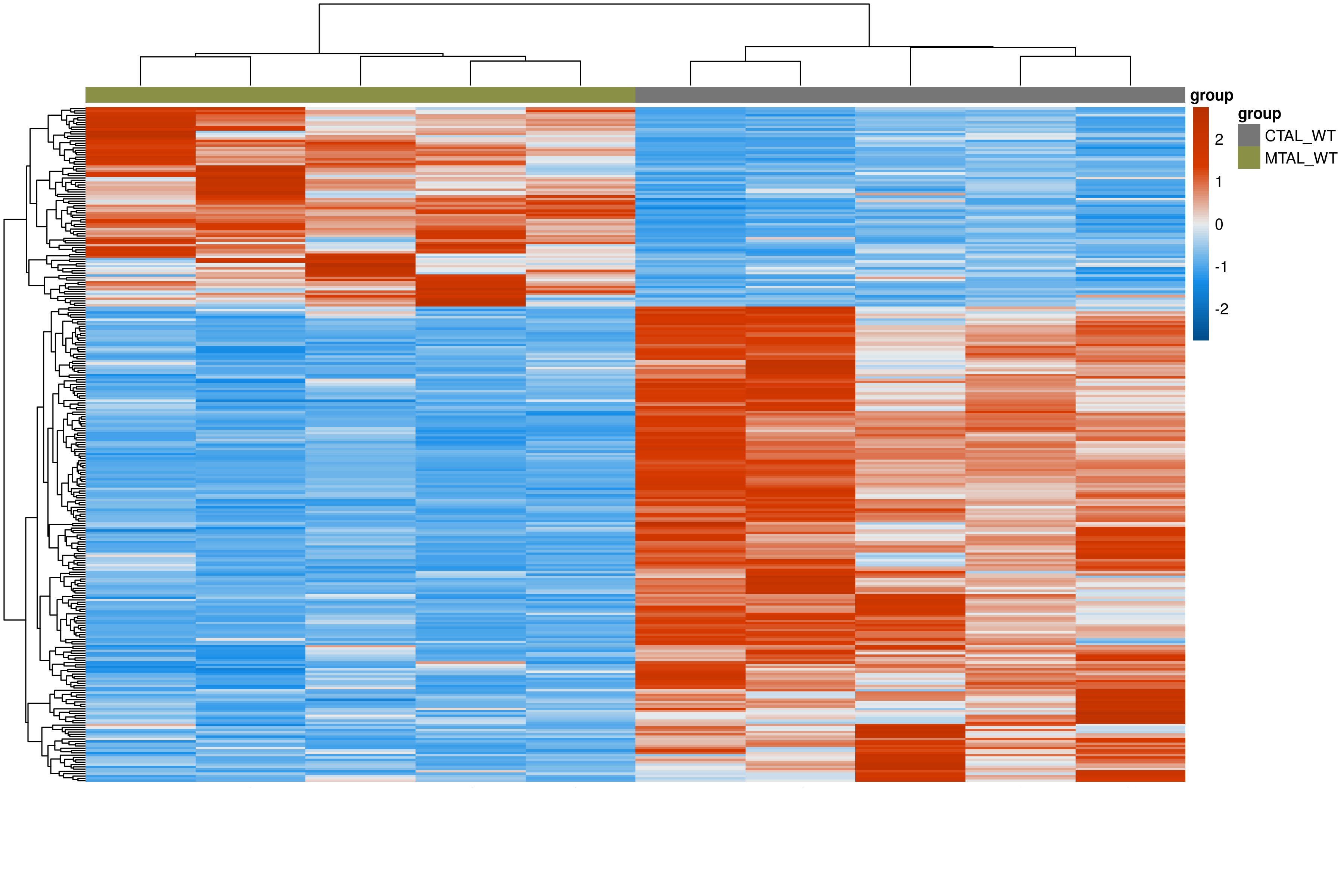

Supplement: Supplementary file 1 [file ijms-25-04008-s001.zip › Prot-Bertoye supplementary material/Supplemental Figure S7 WT MTAL vs WT CTAL global heatmap.tiff]

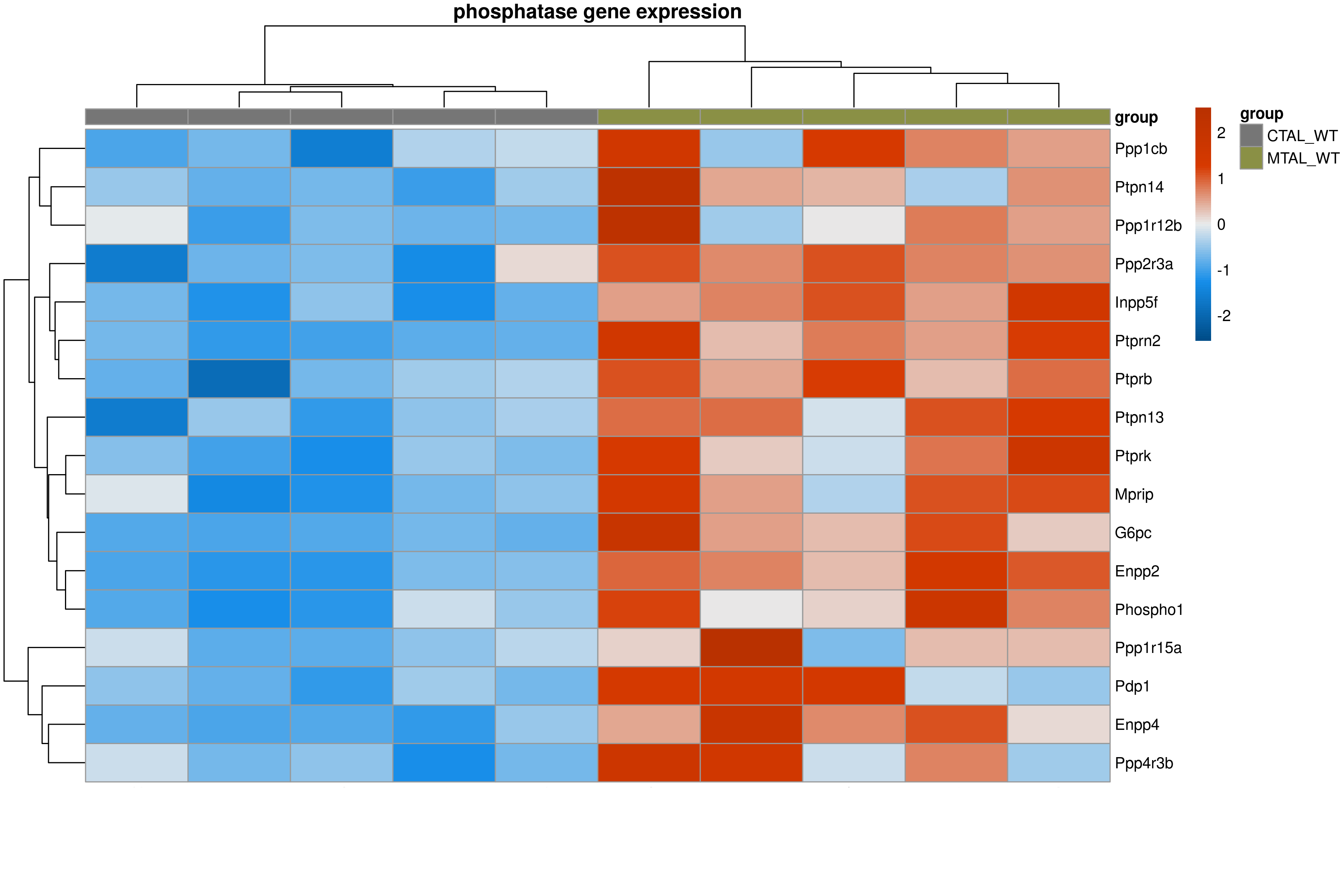

Supplement: Supplementary file 1 [file ijms-25-04008-s001.zip › Prot-Bertoye supplementary material/Supplemental Figure S8 WT MTAL vs WT CTAL phosphatase.tiff]

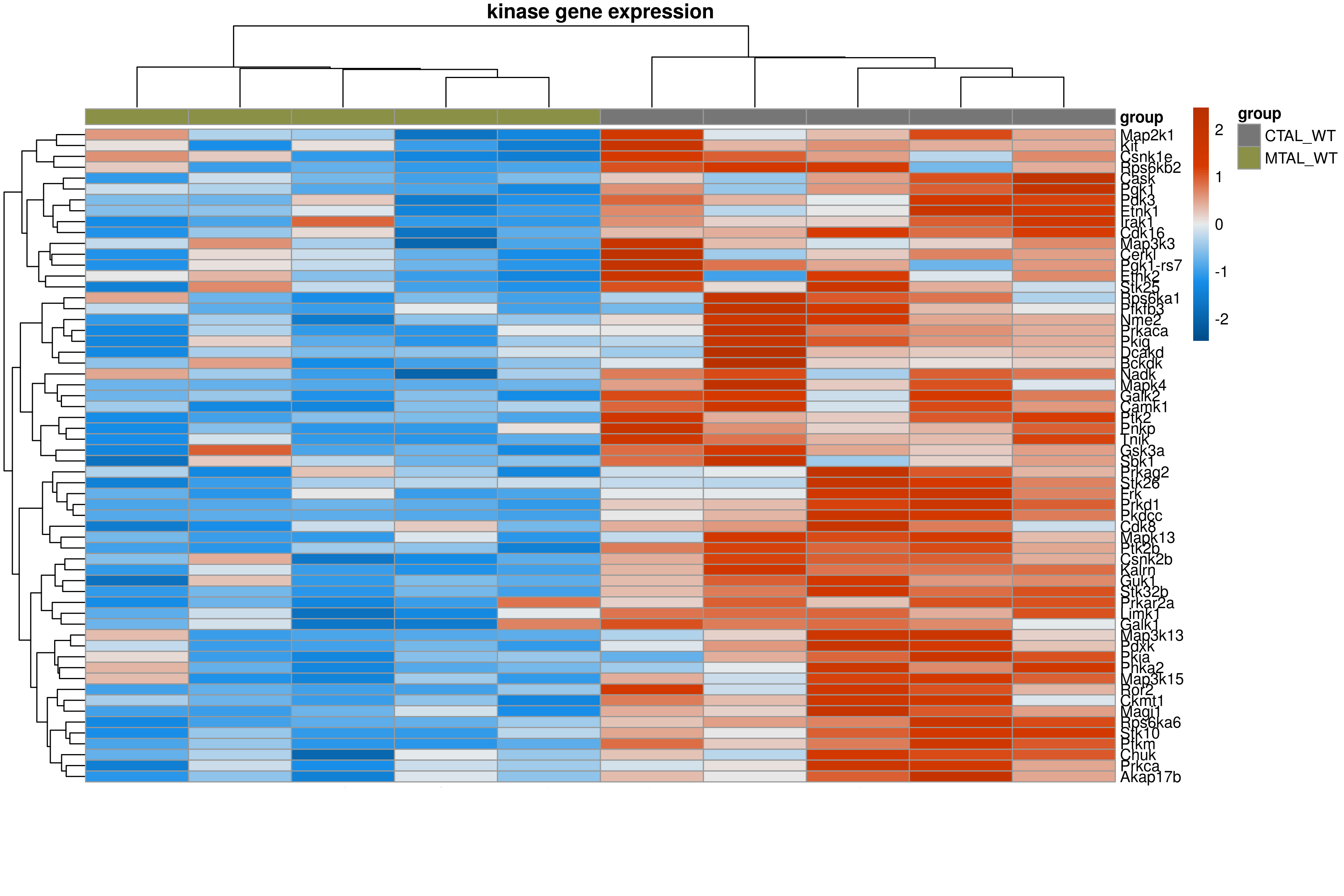

Supplement: Supplementary file 1 [file ijms-25-04008-s001.zip › Prot-Bertoye supplementary material/Supplemental Figure S9 WT MTAL vs WT CTAL kinase.tiff]
